# Pulse-Induced Dynamics of a Charge-Transfer Complex from First Principles

**DOI:** 10.1021/acs.jpca.3c03709

**Published:** 2023-10-12

**Authors:** Matheus Jacobs, Jannis Krumland, Ana M. Valencia, Caterina Cocchi

**Affiliations:** † Physics Department and IRIS Adlershof, Humboldt-Universität zu Berlin, Berlin 12489, Germany; ‡ Institute of Physics, Carl von Ossietzky Universität Oldenburg, 26129 Oldenburg, Germany; ¶ Center for Nanoscale Dynamics (CeNaD), Carl von Ossietzky Universität, Oldenburg 26129, Germany

## Abstract

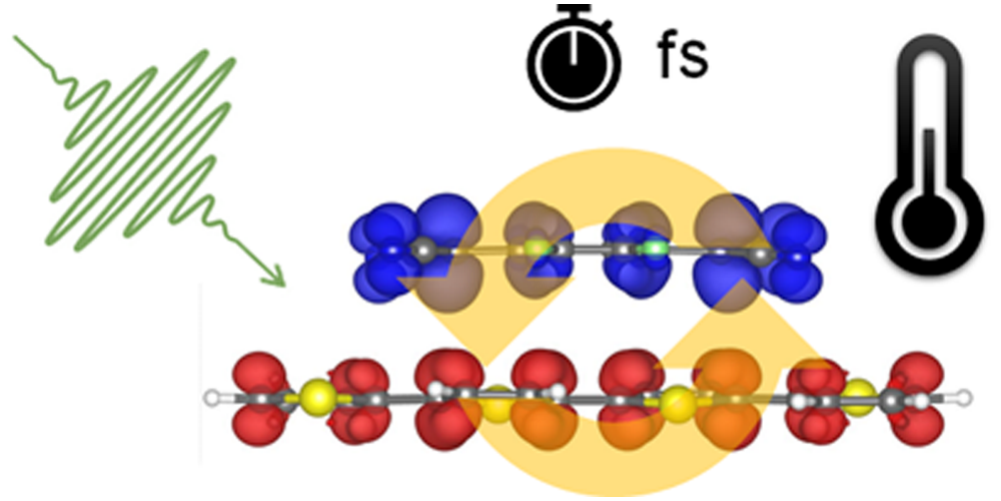

The ultrafast dynamics of charge carriers in organic donor–acceptor interfaces are of primary importance to understanding the fundamental properties of these systems. In this work, we focus on a charge-transfer complex formed by quaterthiophene p-doped by tetrafluoro-tetracyanoquinodimethane and investigate electron dynamics and vibronic interactions also at finite temperatures by applying a femtosecond pulse in resonance with the two lowest energy excitations of the system with perpendicular and parallel polarization with respect to the interface. The adopted *ab initio* formalism based on real-time time-dependent density-functional theory coupled to Ehrenfest dynamics enables monitoring the dynamical charge transfer across the interface and assessing the role played by the nuclear motion. Our results show that the strong intermolecular interactions binding the complex already in the ground state influence the dynamics, too. The analysis of the nuclear motion involved in these processes reveals the participation of different vibrational modes depending on the electronic states stimulated by the resonant pulse. Coupled donor–acceptor modes mostly influence the excited state polarized across the interface, while intramolecular vibrations in the donor molecule dominate the excitation in the orthogonal direction. The results obtained at finite temperatures are overall consistent with this picture, although thermal disorder contributes to slightly decreasing interfacial charge transfer.

## Introduction

The photophysics of organic donor–acceptor complexes is an attractive field of research to unfold the potential of these systems as active components for optoelectronic devices.^1−7^ The complex landscape of often intertwined electronic, optical, and vibrational excitations characterizing this material class depends crucially on the structure–property relationships of the specific systems^4,8−11^ and on the characteristics of the building blocks.^12−14^ Different doping mechanisms emerge according to the electronic interactions between donor and acceptor molecules and crucially influence the response of the complex to external stimuli.

The creation of an anion and a cation upon photoexcitation, known as ion pair formation,^12^ is an effective way to transfer charge carriers across the interface.^12,15^ Examples of such systems are p-doped thiophene polymers,^15,16^ which are often used as active materials in organic solar cells.^17^ Another class of donor–acceptor compounds is given by so-called charge-transfer complexes (CTCs).^12,15^ These doped systems are characterized by fractional charge transfer in the ground state accompanied by electronic hybridization between the frontier orbitals of the two molecular species.^8,15,18−20^ In the excited state, different types of excitations can be formed, again, depending on the characteristics of the building blocks^20^ and on the local interfaces between them^21^ even in the crystalline phase.^22^ Notably, in CTCs, the lowest energy excitation is bright owing to the non-negligible overlap between the bonding and antibonding frontier orbitals.^15,20,21^

Understanding the excitation dynamics of donor–acceptor complexes is of prime importance to clarify the fundamental processes ruling photoabsorption, charge separation, and diffusion:^23−25^ this is essential information to predict the performance of these systems in optoelectronic devices and solar cells.^26−28^ Exploring the ultrafast regime of excitation in the natural, subpicosecond time scale of electrons gives insight into the charge-transfer mechanisms driven by resonant photon absorption.^29^ The coherent coupling between electronic and vibrational degrees of freedom was identified as the key mechanism driving the separation of photoexcited charge carriers across the interface of a noncovalently bound donor–acceptor blend.^24,30−34^ Moreover, time-resolved Raman spectroscopy studies revealed the relevance of vibronic motion in driving chemical reactions and electron transfer in donor–acceptor compounds.^35,36^ Stimulated by these findings, it is interesting to turn to CTCs and try to answer fundamental questions for such systems characterized by frontier-orbital hybridization and fractional charge transfer in the ground state. Addressing (i) the characteristics of ultrafast charge transfer upon the resonant excitation of different electronic transitions, (ii) the role of vibronic coupling, particularly, which vibrational modes come into play under specific excitation conditions, and (iii) the effects of temperature on the coherence of the process is of primary importance to gain deeper understanding on the intrinsic light–matter coupling processes occurring in this class of organic materials.

In this work, we focus on the prototypical CTC formed by a quaterthiophene (4T) molecule p-doped by the strong electron acceptor 2,3,5,6-tetrafluoro-7,7,8,8-tetracyanoquinodimethane (F4TCNQ). We investigate the charge-carrier dynamics in this system, including the nuclear motion, adopting state-of-the-art first-principles methods. In the framework of real-time time-dependent density-functional theory coupled with the Ehrenfest scheme, we monitor the distribution of the electron density across the resonantly excited interface in a 100 fs time window. We analyze the vibrational modes that participate in the dynamics and explore temperature effects adopting thermalized ensembles from Born–Oppenheimer molecular dynamics simulations. Our results reveal substantially different scenarios depending on the polarization of the resonant radiation in both the electronic and the vibronic dynamics. The excitation of the transition between the bonding and antibonding frontier orbitals leads to pronounced oscillations of the charge distribution. The charge density sloshes between the donor and the acceptor molecule with the participation of coupled vibrational modes of both 4T and F4TCNQ. In contrast, the resonant stimulation of the second excited state of the system, corresponding to an optical transition polarized perpendicular to the interfacial direction, is dominated by the dynamics in 4T. In this scenario, the charge distribution does not slosh between donor and acceptor but under the simulated conditions, charge transfer does not effectively increase with respect to the ground state either. At finite temperatures, disorder effects become prominent: the linear absorption spectrum becomes broader and the external pulse leads to the (partly off-resonant) activation of the first and second excitations simultaneously. The results obtained in the adopted formalism, where quantum effects are accounted for only for the electronic system, point to an overall decrease in charge transfer compared to the ground state.

## Methods

### Theoretical Background

The results presented in this work are obtained from first principles in the framework of real-time time-dependent density-functional theory (RT-TDDFT). In this approach, the time-dependent Kohn–Sham (TDKS) equations,^37^
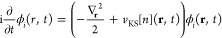
1are solved directly through their propagation in real time. In eq 1, ϕ_*i*_(**r**, *t*) are the wave functions that are used to compute the time-dependent density, *n*(**r**, *t*) = ∑_*i*_*P*_*i*_|ϕ_*i*_(**r**, *t*)|^2^, where *P*_*i*_ is the population of the *i*-th state. The central quantity in eq 1 is the TDKS potential defined as
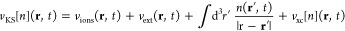
2where *v*_ions_(**r**, *t*) describes the interactions between electrons and nuclei, *v*_ext_(**r**, *t*) is the external time-dependent potential, the integral is the Hartree potential, and *v*_xc_[*n*](**r**, *t*) corresponds to the exchange–correlation (XC) potential. To describe electron–nuclear couplings, RT-TDDFT is interfaced with the Ehrenfest scheme, a nonadiabatic approach for molecular dynamics^38,39^ where the nuclei are treated classically and average electrostatic forces are calculated for the electrons as
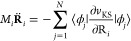
3where ϕ_*j*_ are the solutions of eq 1, *M*_*i*_ is the mass of the *i*-th nucleus, and **R**_*i*_(*t*) is its trajectory. The solution of eq 3 allows monitoring the kinetic energy of each normal mode of the system through the relation
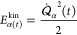
4where  is the time derivative of the normal coordinate associated with the vibrational mode α.

To explore the dynamics in different thermal configurations, we performed Born–Oppenheimer molecular dynamics (BOMD) simulations.^40^ In this scenario, the system is coupled to a thermal bath described by the Nose thermostat^41,42^ until thermalization is reached for each target temperature (100, 200, and 300 K). We choose such a thermostat to ensure proper sampling of the canonical ensemble with thermal fluctuations.^43,44^ No quantum effects are taken into account in the molecular dynamics calculations. While this is a common strategy also consistent with the classical approximation taken for the nuclei, we consider that this can be expected to somewhat underestimate the effect of nuclear motion on the electronic dynamics due to the lack of zero-point energy.^45^ However, we do not anticipate this to influence our results on a qualitative level. Another caveat concerns the inability of Ehrenfest dynamics to describe wave packet splitting. As a consequence, results can become inaccurate if excited-state dynamics are governed by potential-energy surfaces that differ qualitatively from the ground-state one, e.g., that do not support the bound nuclear motion and would lead to dissociation. Here, we checked that no substantial reconfiguration occurred between the molecules, which remain in a face-to-face mutual arrangement due to their strong coupling in the CTC.

To sample uncorrelated snapshots, we compute the autocorrelation function of the energy as
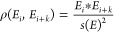
5where *E*_*i*_∗*E*_*i*+*k*_ represents the convolution between energies *E*_*i*_ and *E*_*i*+*k*_, *s*(*E*) is the energy variance, and *k* is the time step between two consecutive samples. The snapshots are sampled at intervals based on limρ(*E*_*i*_, *E*_*i*+*k*_) → 0, which can be interpreted as the time required by the system to lose the memory of its previous configuration. Corresponding positions and velocities are used to initialize the RT-TDDFT simulations at each temperature. The time evolution of different quantities *X* ensuing laser excitation is averaged across the ensemble. The result is closely linked to the time-dependent dipole-*X* correlation function, since the laser is coupled to the system in the dipole approximation.

Linear-response TDDFT calculations^46,47^ are performed to analyze the character of the optical excitations, including their polarization, oscillator strengths, and composition. In this framework, the transition density is computed as
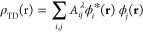
6where *A*_*ij*_^λ^ are the coefficients associated with each single-particle transition contributing to the excitation λ. eq 6 provides a visual representation of the orientation of the transition dipole moment of each excitation. Density-functional perturbation theory calculations^48,49^ were carried out to compute vibrational frequencies and normal modes in the ground-state electronic configuration.

### Computational Details

RT-TDDFT+Ehrenfest calculations are performed using the OCTOPUS code.^50^ The FIRE algorithm^51^ is employed to relax the initial structure until the residual forces are smaller than 10^–3^ eV/Å. For the ground-state calculations, a real-space grid with spacing 0.11 Å is adopted in a simulation box formed by interlocked spheres of radius 6.0 Å centered on each atom. Hartwigsen–Goedecker–Hutter norm-conserving pseudopotentials simulate core electrons.^52,53^ The local-density approximation (LDA) in the Perdew–Zunger parametrization^54^ is used for the XC potential. We checked that this functional is able to deliver a qualitatively correct description of the linear absorption properties of the considered CTC in comparison with many-body perturbation theory calculations.^20^ Also, the adiabatic LDA in combination with Ehrenfest dynamics offers a reliable and affordable way to compute vibronic dynamics^23,24,55^ in spite of its known limitations.^56−58^ We use the term “vibronic” herein, implying with it the coupling between optically driven electronic excitations and vibrational motion with the latter computed at the level of classical molecular dynamics, as discussed above. This being said, we acknowledge that part of the community uses this term primarily in the context of electron-vibration coupling problems where nuclei are treated at the quantum level.^59^

To compute the linear absorption spectrum according to the Yabana–Bertsch scheme,^60^ the KS orbitals obtained by solving eq 1 are propagated through the approximated enforced time-reversal symmetry (AETRS) propagator^61^ with a time step of 0.37 as and a total simulation duration of 20 fs. A “kick” of magnitude 0.0053 Å^–1^ is applied in all Cartesian directions to trigger the time propagation. The grid parameters are the same as in the ground-state calculations. Absorption spectra computed at finite temperatures are averaged over 100 configurations. In the ultrafast dynamics simulations at 0 K, a time-dependent electric field is applied in resonance with two selected excitations giving rise to absorption maxima at 1.2 and 1.5 eV in the linear absorption spectrum. The applied laser pulse of Gaussian shape is centered at 8 fs and has a standard deviation of 2 fs. The peak intensity is set to 300 GW/cm^2^ in order to simulate the response of the system to a strong laser, thus mimicking conditions similar to those achieved in specialized laboratories.^62,63^ Such intensities are typically adopted in RT-TDDFT simulations of organic, inorganic, and hybrid materials^55,64−67^ In previous work on an organic/inorganic interface,^68^ we found that the same intensity ensures effective excitation without inducing sizable nonlinear effects or the ionization of the system. On the other hand, we checked that adopting a much weaker pulse may give rise to spurious numerical noise, which, in turn, may affect the quality of the results and the reliability of their interpretation. We emphasize that by exciting the system with a resonant pulse, we aim to investigate the effects of electronic coherence on the nuclear motion of the CTC. Such a scenario cannot be realized if the system is initially prepared in an excited state.^69−71^ We emphasize that our simulations do not target sunlight harvesting or photosynthesis, which are driven by incoherent and broadband radiation of much lower intensity compared to the one applied here.

BOMD simulations are performed with the CP2K package.^72^ The computational parameters adopted in this step are the same as for the RT-TDDFT calculations, with the exception of the adopted basis set (DZVP^73^) including a plane-wave cutoff of 600 Ry. In this analysis, we considered finite temperatures of 100, 200, and 300 K for which thermal effects are expected to be sizable; lower temperatures, for which quantum nuclear effects are known to be even more prominent than at room temperature,^74,75^ are deliberately excluded from this investigation. During thermal equilibration at 100, 200, and 300 K, a heating ramp drives the system smoothly to the target temperature in the canonical ensemble. To sample structures without any external constraints, at each temperature the system is propagated for 3 ps in the microcanonical ensemble (*NVE*). Each snapshot obtained from this preprocessing is then propagated for 100 fs in the framework of RT-TDDFT+Ehrenfest (AETRS propagator) adopting the OCTOPUS code and the same parameters listed above. To evaluate charge transfer, the density is output every 2 fs and the Bader charges are calculated^76^ averaging over 100 different structures in each thermal ensemble.

## Results and Discussion

### Ground-State Charge Transfer and Linear Absorption

4T-F4TCNQ is a CTC characterized by fractional charge transfer in the ground state.^15,20^ This behavior is induced by the strong p-doping character of F4TCNQ, which according to our results (see Table 1) withdraws more than 0.6 electrons from the donor. This estimate is in line with reference values in the literature obtained at analogous levels of theory;^21^ quantitative differences are ascribed to the adopted approximations (basis sets, XC functional, etc.). Structural details in the construction of the complex and in particular local interfaces between donor and acceptor molecules may play a role, too, as extensively discussed in ref (21). However, it is generally accepted that the bimolecular cluster considered in this analysis is a reasonable model for 4T-F4TCNQ pseudocrystals and blends.^15,77^ We acknowledge, though, that long-range effects may not be captured properly in such a configuration^22^ and the extent of charge delocalization may be underestimated with respect to experiments. However, measurements for the specific system and conditions under consideration are currently unavailable and, thus, a direct assessment is not possible to date. Examining the values reported in Table 1 for finite temperatures, we notice that fluctuations with respect to the result at 0 K are below 10%. The largest variation, on the order of 0.05 e, is seen at 100 K. At higher temperatures, the amount of charge transferred decreases again, reaching very similar values (0.654 and 0.651 e) at 200 and 300 K, respectively. This behavior suggests that changes with respect to the 0 K scenario are induced by the thermal fluctuations that are naturally present in the simulations. However, the overall charge transfer, which is driven by the local interactions between the donor and the acceptor species,^21^ is robust enough to be almost independent of temperature.

**Table 1:** Absolute Value of Charge Transfer and Standard Deviation in the 4T-F4TCNQ Complex Computed at Each Temperature Using the Bader Scheme Averaging over 100 Different Structures

temperature (K)	charge transfer (e)
0	0.622
100	0.674 ± 0.002
200	0.654 ± 0.001
300	0.651 ± 0.003

**Figure 1:**
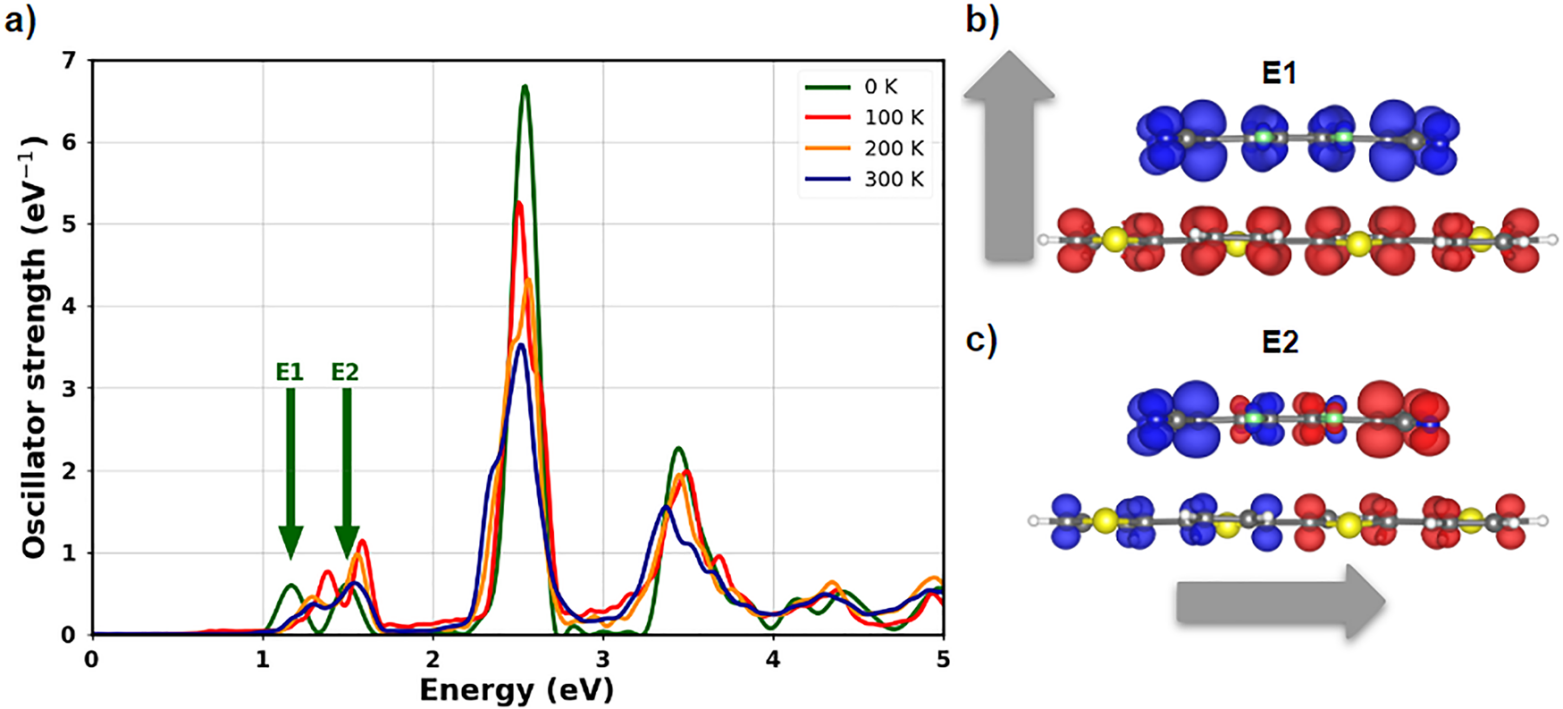
(a) Linear absorption of 4T-F4TCNQ computed at increasing temperatures. Transition densities associated with (b) the first excitation (E1) and (c) the second excitation (E2) indicated in the spectrum at 0 K in panel a. Blue and red isosurfaces indicate domains of charge depletion and accumulation, respectively. The gray arrows highlight the direction of the transition dipole moment.

In the next step of our analysis, we inspect the linear absorption spectrum of the 4T-F4TCNQ complex. The result obtained at 0 K (Figure 1a) is computed from RT-TDDFT in the adiabatic local density approximation. For this reason, excitation energies and relative oscillator strengths differ quantitatively with respect to the many-body perturbation theory results of refs (20) and (21). Yet, the main characteristics of those spectra are retained: two weak excitations appear in the low-energy region followed by more intense resonances at higher energies. The first two maxima, labeled E1 and E2 in Figure 1a, correspond to Frenkel excitons in the whole complex.^20,21^ This nomenclature is adopted based on the evidence that the corresponding electron and hole densities are delocalized on both donor and acceptor molecules, as extensively discussed in refs (20) and (21). The spatial distribution of the electron–hole pairs is directly related to the orbitals contributing to them.^20^ E1 stems from the transition between the highest occupied molecular orbital (HOMO) and the lowest unoccupied one (LUMO), bearing bonding and antibonding character (see Figure S2), respectively;^15,20^ as such, E1 is polarized across the donor/acceptor interface as illustrated by the transition density shown in Figure 1b. We stress that the color domains in those plots refer to positive and negative values of the transition dipole moment, not to the distribution of electrons and holes. Conversely, E2 corresponds to a transition between the HOMO–1 and the LUMO,^20^ with the resulting dipole moment parallel to the long molecular axis (see Figure 1c). The intense maximum centered at about 2.5 eV (see Figure 1a) corresponds to an excitation where both the hole and the electron are localized on the donor. Given their complementary characteristics, E1 and E2 are most suited to be targeted in the analysis of resonantly driven charge-transfer dynamics.

Before moving on in this direction, it is instructive to clarify the influence of temperature on the linear absorption spectrum of 4T-F4TCNQ. The results reported in Figure 1a indicate that the primary effect is a smearing of the absorption peaks, which become less intense and undergo a redistribution of their spectral weight over a wide energy range. This broadening is related to the average performed over of 100 different configurations in the thermalized ensembles. Additionally, at finite temperatures, E1 is blue-shifted by a few hundred meV compared to its counterpart at 0 K. This behavior can be explained in terms of energetic decoupling of the HOMO and the LUMO of the complex: when temperature increases, the donor and acceptor molecules increase their mutual distance by about 0.2 Å compared to the 0 K scenario (see Figure S5). As a result, the frontier levels are shifted to lower and higher energies, giving rise to the increase of the optical gap testified by the blue shift of E1 (HOMO → LUMO transition). Considering that the energy of E2 (HOMO–1 → LUMO) increases with temperature by only a few hundred meV (Figure 1a), we can infer that the HOMO is more susceptible to thermal effects than the LUMO. In the spectra computed at finite temperatures, the blue shift of both peaks, combined with the enlarged smearing due to ensemble averaging, gives rise to a broad absorption maximum including both E1 and E2 (see Figure 1a). At higher energies, the effect of temperature is less dramatic. This can be explained by the fact that the intense excitation at about 2.5 eV corresponds to an intramolecular transition in the donor:^78^ the involved orbitals are localized on 4T and, as such, are only marginally affected by the structural reorganization of the complex on account of thermal effects.

### Charge-Transfer Dynamics and Vibronic Coupling

**Figure 2:**
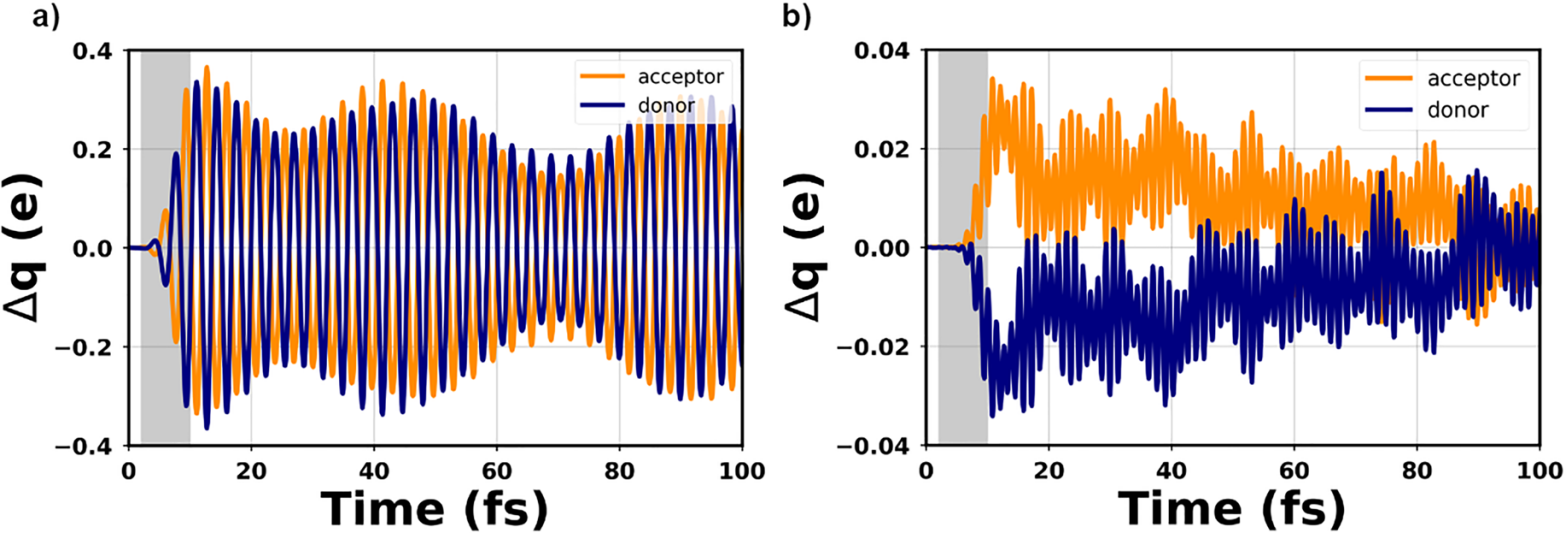
Time evolution of the partial charges, computed with respect to the ground-state value, on the donor (4T) and acceptor molecule (F4TCNQ) of the complex upon the resonant excitation of (a) E1 and (b) E2. The gray, shaded area indicates the time window in which the time-dependent pulse is active.

As a first step in the analysis of the charge-transfer dynamics of the CTC, we inspect the time evolution of the partial charge distribution across the interface with respect to the reference ground-state values reported in Table 1. This quantity represents the time-dependent quantum-mechanical expectation value of the charge transfer in the prepared superposition state. By stimulating E1 at 0 K, the partial charge variation, Δ*q*, exhibits a pronounced oscillatory behavior with a large amplitude across negative to positive values (Figure 2a). The oscillation period, which remains essentially constant throughout the entire simulation window, is of approximately 3.45 fs, matching the energy of the incident pulse (1.2 eV). This behavior indicates that the resonant pumping of E1 leads to an electronic charge sloshing across the interface that does not ultimately lead to charge separation in the considered 100 fs time window. Vibronic motion, which is responsible for the amplitude modulation of Δ*q*, cannot revert the mentioned trend.

The charge-transfer dynamics obtained by resonantly exciting E2 differ both qualitatively and quantitatively from the above-discussed results for E1 (Figure 2b). In the first 50 fs, Δ*q* is positive on the acceptor and negative on the donor, suggesting an enhancement of charge transfer driven by the applied resonant field. After about 80 fs, the partial charges on the donor (acceptor) start assuming negative (positive) values; around 100 fs, the baseline of both trends is very close to the ground-state value. In the stimulation of E2, electron-vibrational couplings do not give rise to a clear amplitude modulation of Δ*q*, as seen instead for E1. Furthermore, we note that the absolute values of Δ*q* in Figure 2a and Figure 2b differ from each other by 1 order of magnitude.

Before continuing with the analysis, it is worth commenting on the results obtained so far in comparison with similar studies. In refs (23) and (24), it was demonstrated that vibrational coherence enhances charge transfer. However, the systems investigated in those works are intrinsically different from the one explored here. The donor–acceptor complex addressed in ref (24) does not exhibit sizable charge transfer nor orbital hybridization in the ground state. The creation of an electron–hole pair in one subsystem leads to charge separation upon the action of an external field and is assisted by vibrational motion. Conversely, in the CTC examined here, the targeted excitations E1 and E2 are both delocalized on the entire complex (see Figure 1b,c and refs (20) and (21)) and are thus “charge transfer” in nature. The application of a pulse in resonance with them triggers and enhances the charge transfer with respect to the ground state with a marginal role of vibrations compared to the case discussed in ref (24). Also, the dynamics of E2 resemble the one obtained at the same level of theory for a prototypical hybrid inorganic/organic interface in which F4TCNQ p-dopes an H-terminated Si cluster,^67^ where the pumped excitation has also perpendicular polarization with respect to the interfacial direction; i.e., the transition dipole moment is parallel to the long molecular axis. Finally, the results reported in Figure 2 indicate that the laser-excited CTC remains in a coherent superposition between the ground and the excited state, as expected from the adopted RT-TDDFT+Ehrenfest scheme.^55^ As such, quantities of interest in the context of quantum-chemical studies on transition barriers, such as reorganization energies,^79−81^ are not particularly meaningful in this context.

To understand the dynamics reported in Figure 2, it is useful to deepen the analysis on the vibronic coupling of the 4T-F4TCNQ complex. Specifically, since both E1 and E2 are intermolecular excitations (Figure 1b,c), it is relevant to understand whether the vibrational modes of the acceptor and donor interact in the ground state, and if they are activated by laser excitation. To answer these questions, we analyze the vibrational spectrum of the complex and find three modes, with frequencies 1403, 1430, and 1446.5 cm^–1^, coupling the C=C stretching in F4TCNQ (bonds connecting the tetrafluorinated phenyl rings with the cyano groups) with the so-called C_α_–C_β_ mode^82^ in 4T. In the following, we will refer to them as Q_*a*_, Q_*b*_, and Q_*c*_, respectively (see Figure S3). We recall that the adopted RT-TDDFT+Ehrenfest formalism includes nonadiabatic effects that are expected to be important in these dynamics.

**Figure 3:**
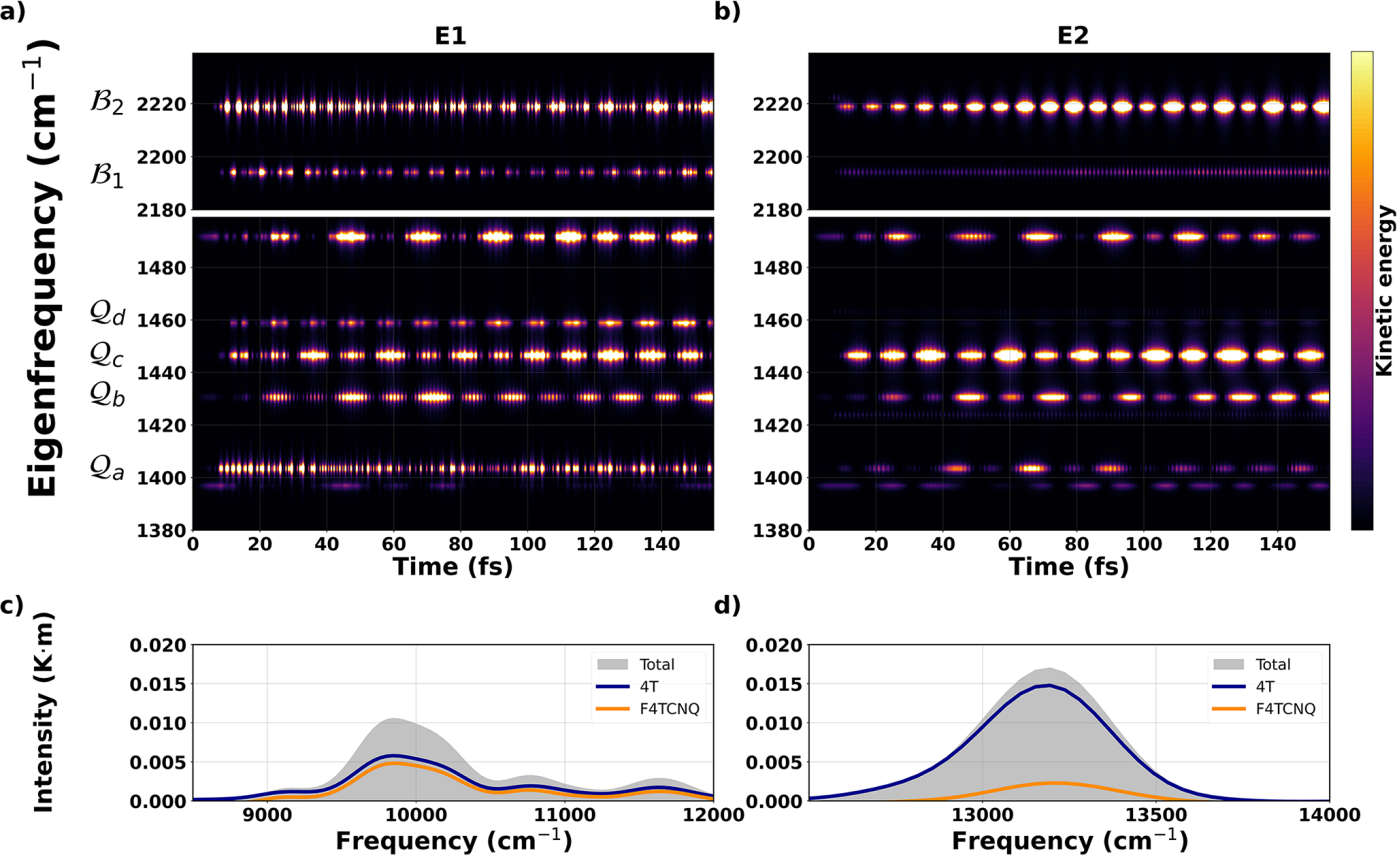
(a) Kinetic energy of the vibrational modes during the laser-induced dynamics for E1. (b) Kinetic energy of the vibrational modes during the laser-induced dynamics for E2. Power spectra of the CTC (gray area) upon the excitation of (c) E1 and (d) E2, with the contributions of the constituents (4T and F4TCNQ) marked by continuous lines.

Following the strategy adopted in previous work,^83^ we examine the kinetic energy accumulated by these modes during laser irradiation and the subsequent evolution of the vibronic degrees of freedom in the 4T-F4TCNQ complex. In Figure 3a, we show a map of the kinetic energy of the normal modes during the time evolution of the system excited in resonance with E1. All coupled modes, Q_*a*_, Q_*b*_, and Q_*c*_, undergo an increase in the kinetic energy; the period of their oscillation is about 3.40 fs, which corresponds to the one of the incident pulse. We interpret this result as follows. The laser polarization is perpendicular to the conjugated backbone of the molecules and hence to the covalent bonds therein. The electronic density is consequently perturbed and induces a force that drives the oscillation of the electrons in the system at the field frequency. This force interacts with the harmonic motions of some of the modes, e.g., Q_*b*_, and Q_*c*_, causing their oscillation at the sum of the natural and the field frequency. We will come back to this point later on; for the analytic derivation of this force, see the Supporting Information. Also, it is worth highlighting that the perpendicularly polarized pulse in resonance with E1 weakly activates a normal mode in F4TCNQ at 1458 cm^–1^ (Q_*d*_ in Figure 3); the same does not happen for a pulse with parallel polarization such as the one stimulating E2 (cf. Figure 3a,b). A similar behavior, although less pronounced, is exhibited also by the C≡N modes appearing at frequencies above 2000 cm^–1^ and labeled as B_1_ and B_2_ in Figure 3a; for their visualization, see Figure S4.

Upon the excitation of E2, a different scenario is disclosed, see Figure 3b. A significantly larger amount of kinetic energy with respect to the irradiation of E1 is accumulated by Q_*c*_, while the behavior of Q_*b*_ is essentially the same in the two cases. Moreover, it can be seen that upon resonant excitation of E2, the C≡N mode at 2218 cm^–1^ (B_2_) gains kinetic energy throughout the whole simulation window in a more consistent and pronounced way than upon the excitation of E1. We explain this behavior considering that the C≡N bond lies on the plane of the acceptor molecule, and thus, it is aligned with the polarization of the external field in resonance with E2. For completeness, it is worth specifying that in 4T-F4TCNQ, the acceptor molecule is slightly bent, with the N atoms pointing toward the donor. This is a known behavior related to the role of the cyano groups in mediating charge transfer in those organic complexes.^77^

Additional insight can be gained by contrasting the power spectrum of the complex against the contributions of its components. The results displayed in Figures 3c,d represent the amount of energy density accumulated by the system when excited in resonance with E1 and E2, respectively, around the frequency of the corresponding incident pulse (1.2 eV = 9678.65 cm^–1^ for E1 and 1.5 eV = 12098.31 cm^–1^ and for E2). When the complex is pumped at the energy of E1, the power accumulated due to the excitation of the vibrational degrees of freedom is almost evenly distributed between 4T and F4TCNQ (Figure 3c). Notice that, in this case, the power spectrum has maxima not only at the laser frequency but also at higher energies. These additional bands are ascribed to the interaction between the harmonic nuclear motion and the fast oscillating force due to coherence with the field, which is discussed in the Supporting Information.

In the resonant stimulation of E2, on the other hand, the vibronic response of the complex mostly comes from the donor (Figure 3d) with the contribution of the acceptor peaked around the carrier frequency of the incident pulse. We recall that in E2, dominated by the HOMO–1 → LUMO transition, the excited charge density is still delocalized over the whole complex but the excitation is polarized along the long molecular axes.^20,21^ As such, the intramolecular modes couple more efficiently with the electronic dynamics, and the distribution of the kinetic energy is no longer equally spread between the donor and acceptor. As shown in Figure 3b, the coupled normal modes that mostly accumulate kinetic energy upon the excitation of E2 are Q_*c*_ and B_2_. On the other hand, the energy accumulated by Q_*a*_ and Q_*b*_ is much weaker compared to the case in which E1 is stimulated, explaining therefore the asymmetric distribution of kinetic energy in favor of the donor (Figure 3d).

**Figure 4:**
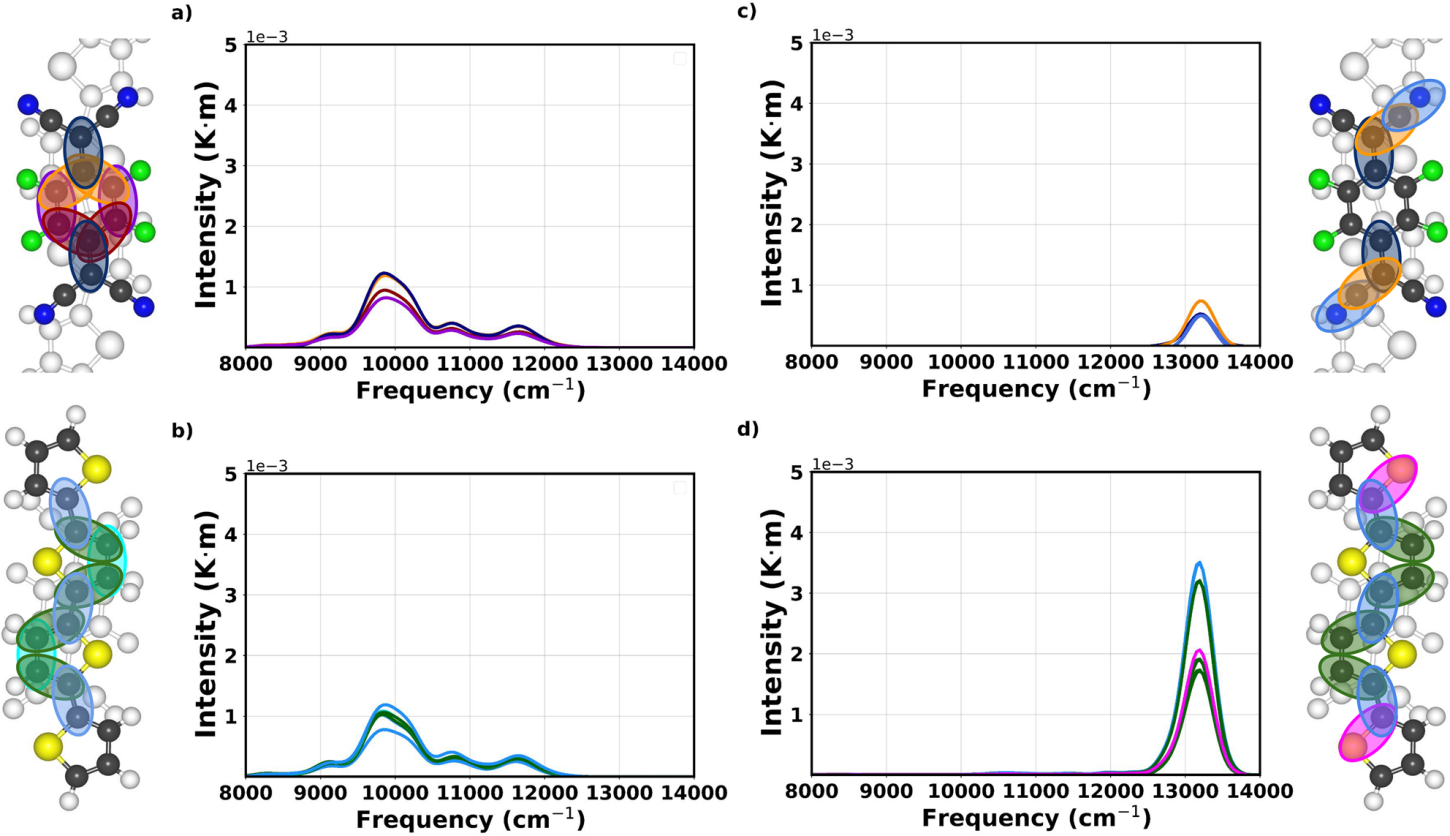
Power spectra associated with the dominant vibrational modes of F4TCNQ (panels a and c) and 4T (panels b and d), when the complex is excited by a laser pulse in resonance with (a,b) E1 and (c,d) E2. The color code for the solid lines in the spectra shows each bond of the complex by symmetry, and they are shown in the ball-and-stick representation of the complex shown on the side of each panel.

For a deeper comprehension of these results, we now inspect the power spectrum associated with the single bond oscillations in the donor and the acceptor molecule of the CTC excited in resonance with E1 and E2. In Figure 4, left (right) panel, results obtained upon the excitation of E1 (E2) are shown; on the top and bottom panel, the contributions of vibrations in F4TCNQ and in 4T are reported, respectively. Upon excitation of E1, the strongest vibronic contribution in the acceptor comes from the C=C mode (Figure 4a). This is not surprising, considering the participation of this oscillation in the coupled modes Q_*a*_, Q_*b*_, and Q_*c*_ (see Figure S3). Additional contributions come from the C–C bonds in the tetrafluorinated phenyl ring. Looking at the response of 4T to the same excitation, we find the strongest response arising from the C_α_–C_β_ and the C_β_–C_β_ bond oscillations (Figure 4b). Notice that among the four most important vibronic contributions shown in Figure 4b, only C–C oscillations appear. Moving now to the right panels of Figure 4, we appreciate the small contribution of the intramolecular modes of F4TCNQ already discussed with reference to Figure 3d. The results shown in Figure 4c underline the relevance of the C=C bond oscillation in the acceptor, accompanied, however, by a strong contribution from the C–C bond connecting the cyano group to the phenyl ring. Notably, the weight of the C ≡N oscillations is larger than the one associated with the phenyl ring. The C_α_–C_β_ and C_β_–C_β_ modes dominate the vibronic response of 4T also upon the resonant excitation of E2 (Figure 4d).

This analysis suggests that the excitation of E1 and E2 by a resonant pulse triggers two different types of vibronic effects. For the latter, it is due to nuclear relaxation within the individual components; for E1, they correspond to forced nuclear oscillations driven by the coherence between the ground state and the excited state, facilitated by the relatively low energy of this electronic excitation.

### Charge-Transfer Dynamics at Finite Temperatures

The analysis performed so far on the laser-induced charge-carried dynamics at 0 K equips us with the necessary knowledge to investigate the dynamics of the CTC at finite temperatures. Due to the smearing of E1 and E2 in the linear absorption of 4T-F4TCNQ (Figure 1a), at 100, 200, and 300 K it is no longer possible to resonantly target the two excitations separately. For this reason, the results presented below for each temperature value are obtained with a single pulse set at the energy of the lowest energy peak in the corresponding spectrum in Figure 1a.

**Figure 5:**
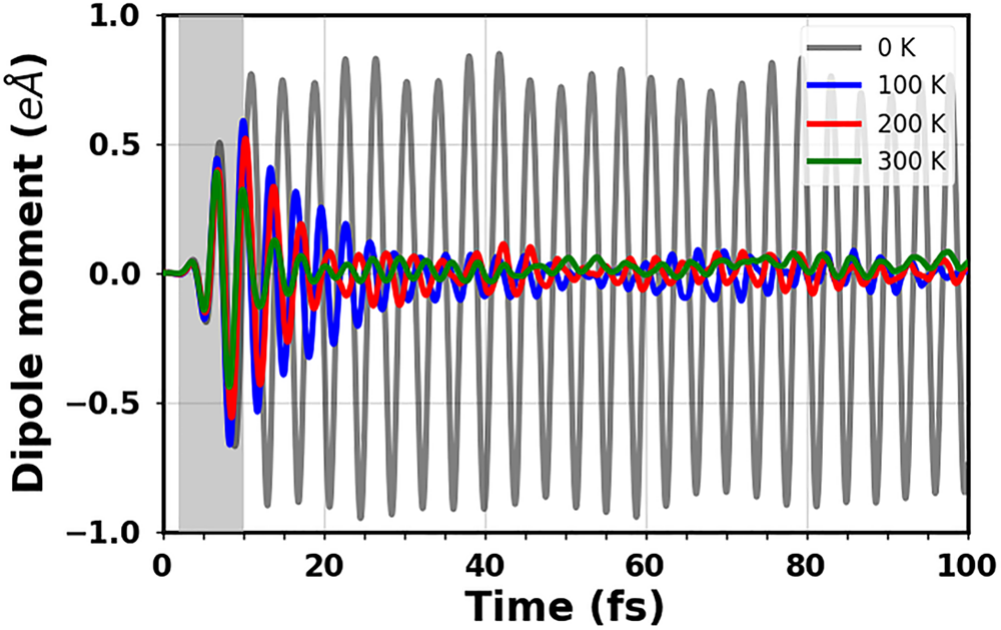
Dipole moment in the *z*-direction induced by the resonant excitation of E1 and averaged over 20 snapshots for each finite temperature. The result at 0 K is shown in gray in the background. The shaded area marks the time window of the pulse.

Before diving into the discussion of dynamical charge transfer at finite temperatures, we make a short digression to inspect the *z*-component (across the interface) of the time-dependent induced dipole moment for each ensemble at 100, 200, and 300 K, and contrast it against the one obtained at 0 K (see Figure 5). At zero temperature (gray line), the dipole moment induced by the stimulation of E1 exhibits large oscillations between positive and negative values in a range between −1 and +1 eÅ over the entire 100 fs window. These persisting oscillations are a signature of coherence in the absence of any dissipation effect. Their quantum-mechanical and electronic nature was demonstrated in previous work^55^ through a direct comparison between RT-TDDFT simulations without nuclear motion and the results of a two-level model. Vibrational effects captured here by the applied Ehrenfest formalism manifest themselves in the amplitude modulation of the dipole moment.

Considering now the results for the ensembles averaged at 100 and 200 K (blue and red curves in Figure 5, respectively), the maximal amplitude is reached at 12 fs, right after the pulse is switched off. However, differently from the result obtained at 0 K, it decays to less than 0.1 eÅ within the first 30 fs. When the temperature increases, each nuclear trajectory remains coherent but the evaluation of the macroscopic polarization as an average across all molecules in the ensemble decays due to thermal fluctuations of the excitation energy.^75^ The dephasing undergone by the induced dipole moment at finite temperatures is influenced by the specific temperature value. In particular, in the ensemble at 300 K (green curve), the maximal amplitude is reached at about 7 fs, when the laser is still on, and drops almost immediately after the switch-off of the pulse. As a note of caution, it is worth specifying that Ehrenfest dynamics does not obey detailed balance.^84,85^ Hence, temperature-induced damping of coherent Ehrenfest dynamics may not necessarily describe the actual physical situation.

**Figure 6:**
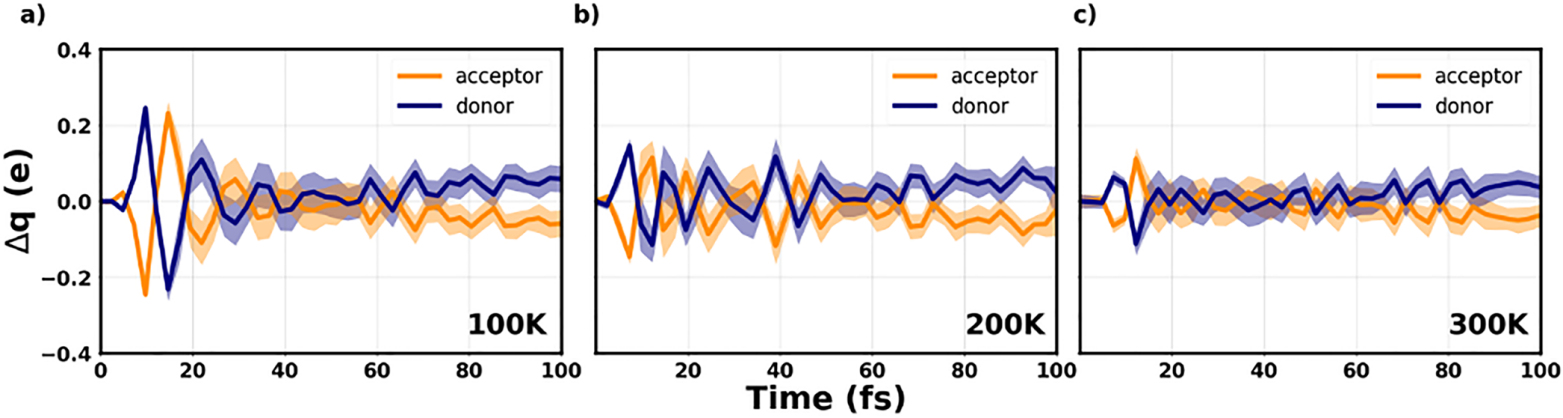
Partial charge variation over time with respect to the ground-state value at the considered temperatures of (a) 100 K, (b) 200 K, and (c) 300 K. The results obtained at each time step are averaged over 20 snapshots and the corresponding uncertainty is visualized by the shaded area surrounding the solid lines.

In the next step, we investigate how temperature affects the dynamical charge transfer in the laser-excited CTC. In analogy with the analysis performed at 0 K, we monitor the temporal variation of the partial charges with respect to the ground-state value obtained at the corresponding temperature (see Table 1). The results shown in Figure 6 indicate an oscillatory behavior of Δ*q*, which assumes positive and negative values both on the donor and on the acceptor throughout almost the entire simulation window. In the first 50 fs, the amplitude of the oscillations decreases systematically as the temperature increases: this suggests not only that the charge transfer is less effective at higher temperatures but also that the system consistently loses coherence. Moreover, it is worth emphasizing once again that at finite temperatures the second excited state, E2, is likely off-resonantly pumped in the stimulation of E1, due to the energetic proximity of these two excitations in the corresponding linear absorption spectra (see Figure 1a). The participation of an off-resonant excitation in the dynamics impacts the coherence. In the second half of the simulation window, the values of Δ*q* are steadily positive on the donor and negative on the acceptor at all considered temperatures (see Figure 6). The time evolution of the resonantly excited thermal ensemble leads to an effective decrease of charge transfer with respect to the corresponding ground-state value. This result can be understood by recalling that the donor and acceptor molecules in the complex are driven slightly apart from each other (Figure S5) as a concomitant effect of the increasing temperature and of the vibronic response to the external field.

**Figure 7:**
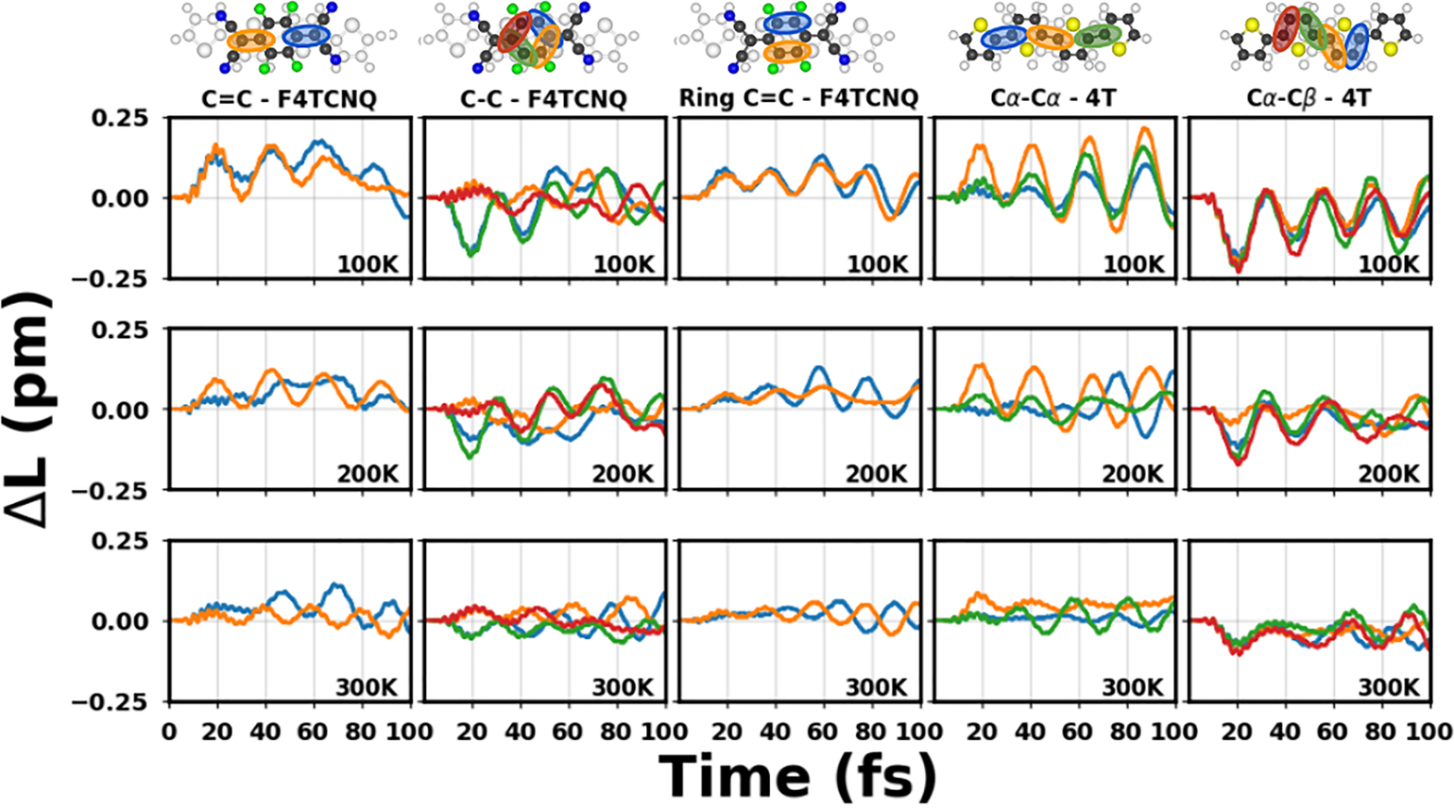
Averaged laser-induced bond length variation (Δ*L*) with respect to the ground state with and without the pulse for each ensemble thermalized at 100, 200, and 300 K. The bonds involved in the dynamics are highlighted in color in the ball-and-stick sketches of the CTC (top).

To investigate these effects more in-depth, we monitor the laser-driven time evolution of the bond lengths involved in the vibrational normal modes of the complex (Figure 7). Let us consider first the dynamics of the C=C bond in the acceptor as a function of temperature. At 100 K, both bond lengths oscillate in phase until 50 fs, when dephasing starts off. At higher temperatures, in-phase C=C oscillations occur only in the first 20 fs; afterward, they begin to dephase. An analogous behavior is seen in the dynamics of the C=C bonds in the phenyl ring of F4TCNQ, as well as in the C_α_–C_α_ and the C_β_–C_β_ oscillations in 4T. Note that all the corresponding modes mostly participate in the coherent charge-transfer process at 0 K (Figures 3 and 4). The C–C bonds in F4TCNQ are less involved in the laser-induced dynamics: they oscillate in phase in the first few femtoseconds but then, at all temperatures, they dephase as the system evolves in time. The decreasing amplitude in the bond length oscillations as a function of temperature is associated with the disorder that occurs when temperature increases. In this case, the system can access different regions of the configurational space, and by doing so, it has different ways to reorganize itself. In other words, more configurations imply higher disorder, while the donor and the acceptor increase their mutual distance as a function of time at finite temperatures (see Figure S5). As seen from Figure 7 and Figure 6, this disorder has a detrimental impact on charge transfer. Averaging over many configurations overall weakens the coupling between 4T and F4TCNQ, and hence their ability to dynamically transfer charge across their interface. We conclude this discussion by recalling that in the adopted formalism, the zero-point energy is neglected. It is known from the literature that overlooking this effect may influence the observed energy flow as well as the thermalization,^86−88^ in particular with regard to the high-frequency modes, which typically are not strongly affected by temperature.

## Conclusions

We have presented a first-principles study on the ultrafast dynamics of an organic complex formed by a 4T molecule p-doped by F4TCNQ. Such a system exhibits a fractional degree of charge transfer in the ground state. We have monitored in time the distribution of the charge carriers following the excitation of the first and second excited states by means of an ultrafast resonant pulse. Stimulating the lowest energy transition between the bonding HOMO and the antibonding LUMO triggers large-amplitude charge oscillations from the donor to the acceptor, consistent with the system being in a coherent superposition between the ground and the excited state. Targeting the second excitation, which is polarized perpendicularly to the interface, i.e., along the long molecular axes, leads to a qualitatively similar to the case of F4TCNQ doping a silicon nanocluster.^67^ The analysis of the vibrations paired with the photoexcited electronic degrees of freedom sheds light on the fundamental mechanisms ruling the two scenarios. We find significant nuclear motion driven by the laser and accompanying harmonic vibrations in spite of the inertia of the heavy nuclei. Coherently excited vibrational modes coupling the donor and the acceptor molecules are responsible for the dynamics of the lowest energy excitation; for the second one, intramolecular bond oscillations, especially in the donor, play a more prominent role. We have completed our study analyzing temperature effects. To do so, we have performed Born–Oppenheimer molecular dynamics simulations to obtain thermalized configurations at 100, 200, and 300 K. In this scenario, we have found disorder effects becoming predominant and effectively decreasing charge transfer with respect to the ground state.

The obtained results provide new insight into the already well-explored area of dynamical charge transfer in organic complexes. Most of the existing work dedicated to the dynamics of organic donor/acceptor complexes was focused on systems forming ion pairs upon photoexcitation. In the latter, the electron–hole pair in one subsystem is separated through the mediation of the vibronic motion. In the complex considered here, instead, charge transfer occurs already in the ground state due to the strong p-doping driven by the acceptor. The frontier orbitals are hybridized with bonding and antibonding character and the transition between them is an actual charge-transfer excitation with the hole and the electron localized on the donor and the acceptor molecule, respectively. In such a scenario, we analyzed the charge-transfer dynamics and the influence of vibrational and thermal degrees of freedom. Due to the peculiar nature of the investigated system, the obtained results may seem to contradict intuition, which is typically built, however, on systems with substantially different characteristics (ion-pair formation upon photoexcitation) and in which charge transfer is driven predominantly by vibronic coherence.^23,24^ With our *ab initio* study, we have explored another scenario and discussed the effects of vibronic and thermal motion therein. We hope that our findings will stimulate corresponding experimental investigations that confirm or challenge them. In parallel, additional theoretical studies adopting more advanced approaches for the nuclear dynamics, accounting, for instance, for quantum nuclear effects^75,89^ may further enhance the understanding of the photophysical properties of charge-transfer complexes subject to intense and coherent irradiation.

## Supporting Information Available

Theoretical analysis and first-principles results (PDF)

## References

[ref1] Schwarz C.; Milan F.; Hahn T.; Reichenberger M.; Kümmel S.; Köhler A. Ground state bleaching at donor–acceptor interfaces. Adv. Funct. Mater. 2014, 24, 6439–6448. 10.1002/adfm.201400297.

[ref2] Hooley E. N.; Jones D. J.; Greenham N. C.; Ghiggino K. P.; Bell T. D. Charge transfer in single chains of a donor–acceptor conjugated tri-block copolymer. J. Phys. Chem. B 2015, 119, 7266–7274. 10.1021/jp510769p. 25417793

[ref3] Fuzell J.; Jacobs I. E.; Ackling S.; Harrelson T. F.; Huang D. M.; Larsen D.; Moulé A. J. Optical dedoping mechanism for P3HT: F4TCNQ mixtures. J. Phys. Chem. Lett. 2016, 7, 4297–4303. 10.1021/acs.jpclett.6b02048. 27731993

[ref4] Neelamraju B.; Watts K. E.; Pemberton J. E.; Ratcliff E. L. Correlation of coexistent charge transfer states in F4TCNQ-doped P3HT with microstructure. J. Phys. Chem. Lett. 2018, 9, 6871–6877. 10.1021/acs.jpclett.8b03104. 30450910

[ref5] Xu W.-L.; Wang Y.-B.; Yang X.-Y.; Qin W.; Hao X.-T. Exploring charge transfer processes and crystallization dynamics in donor-acceptor crystals. Org. Electron. 2018, 58, 105–110. 10.1016/j.orgel.2018.03.037.

[ref6] Cui M.; Rui H.; Wu X.; Sun Z.; Qu W.; Qin W.; Yin S. Coexistent Integer Charge Transfer and Charge Transfer Complex in F4-TCNQ-Doped PTAA for Efficient Flexible Organic Light-Emitting Diodes. J. Phys. Chem. Lett. 2021, 12, 8533–8540. 10.1021/acs.jpclett.1c02281. 34464151

[ref7] Theurer C. P.; Valencia A. M.; Hausch J.; Zeiser C.; Sivanesan V.; Cocchi C.; Tegeder P.; Broch K. Photophysics of Charge Transfer Complexes Formed by Tetracene and Strong Acceptors. J. Phys. Chem. C 2021, 125, 6313–6323. 10.1021/acs.jpcc.0c10815.

[ref8] Salzmann I.; Heimel G.; Duhm S.; Oehzelt M.; Pingel P.; George B. M.; Schnegg A.; Lips K.; Blum R.-P.; Vollmer A.; et al. Intermolecular Hybridization Governs Molecular Electrical Doping. Phys. Rev. Lett. 2012, 108, 035502 10.1103/PhysRevLett.108.035502. 22400758

[ref9] Gao J.; Roehling J. D.; Li Y.; Guo H.; Moulé A. J.; Grey J. K. The effect of 2, 3, 5, 6-tetrafluoro-7, 7, 8, 8-tetracyanoquinodimethane charge transfer dopants on the conformation and aggregation of poly (3-hexylthiophene). J. Mater. Chem. C 2013, 1, 5638–5646. 10.1039/c3tc31047g.

[ref10] Di Nuzzo D.; Fontanesi C.; Jones R.; Allard S.; Dumsch I.; Scherf U.; Von Hauff E.; Schumacher S.; Da Como E. How intermolecular geometrical disorder affects the molecular doping of donor–acceptor copolymers. Nature Commun. 2015, 6, 6460 10.1038/ncomms7460. 25753229

[ref11] Beyer P.; Pham D.; Peter C.; Koch N.; Meister E.; Brütting W.; Grubert L.; Hecht S.; Nabok D.; Cocchi C.; et al. State-of-Matter-Dependent Charge-Transfer Interactions between Planar Molecules for Doping Applications. Chem. Mater. 2019, 31, 1237–1249. 10.1021/acs.chemmater.8b01447.

[ref12] Salzmann I.; Heimel G.; Oehzelt M.; Winkler S.; Koch N. Molecular Electrical Doping of Organic Semiconductors: Fundamental Mechanisms and Emerging Dopant Design Rules. Acc. Chem. Res. 2016, 49, 370–378. 10.1021/acs.accounts.5b00438. 26854611

[ref13] Arvind M.; Tait C. E.; Guerrini M.; Krumland J.; Valencia A. M.; Cocchi C.; Mansour A. E.; Koch N.; Barlow S.; Marder S. R.; et al. Quantitative analysis of doping-induced polarons and charge-transfer complexes of poly (3-hexylthiophene) in solution. J. Phys. Chem. B 2020, 124, 7694–7708. 10.1021/acs.jpcb.0c03517. 32574055

[ref14] Mansour A. E.; Lungwitz D.; Schultz T.; Arvind M.; Valencia A. M.; Cocchi C.; Opitz A.; Neher D.; Koch N. The optical signatures of molecular-doping induced polarons in poly(3-hexylthiophene-2,5-diyl): individual polymer chains versus aggregates. J. Mater. Chem. C 2020, 8, 2870–2879. 10.1039/C9TC06509A.

[ref15] Méndez H.; Heimel G.; Winkler S.; Frisch J.; Opitz A.; Sauer K.; Wegner B.; Oehzelt M.; Röthel C.; Duhm S.; et al. Charge-transfer crystallites as molecular electrical dopants. Nature Commun. 2015, 6, 8560 10.1038/ncomms9560. 26440403 PMC4600739

[ref16] Burke J. H.; Bird M. J. Energetics and Escape of Interchain-Delocalized Ion Pairs in Nonpolar Media. Adv. Mater. 2019, 31, 1806863 10.1002/adma.201806863. 30697829

[ref17] Berger P.; Kim M. Polymer solar cells: P3HT: PCBM and beyond. J. Renew. Sustain. Energy 2018, 10, 013508 10.1063/1.5012992.

[ref18] Aziz E.; Vollmer A.; Eisebitt S.; Eberhardt W.; Pingel P.; Neher D.; Koch N. Localized Charge Transfer in a Molecularly Doped Conducting Polymer. Adv. Mater. 2007, 19, 3257–3260. 10.1002/adma.200700926.

[ref19] Méndez H.; Heimel G.; Opitz A.; Sauer K.; Barkowski P.; Oehzelt M.; Soeda J.; Okamoto T.; Takeya J.; Arlin J.-B.; et al. Doping of Organic Semiconductors: Impact of Dopant Strength and Electronic Coupling. Angew. Chem., Int. Ed. 2013, 52, 7751–7755. 10.1002/anie.201302396. 23784880

[ref20] Valencia A. M.; Cocchi C. Electronic and Optical Properties of Oligothiophene-F4TCNQ Charge-Transfer Complexes: The Role of the Donor Conjugation Length. J. Phys. Chem. C 2019, 123, 9617–9623. 10.1021/acs.jpcc.9b01390.

[ref21] Valencia A. M.; Guerrini M.; Cocchi C. Ab initio modelling of local interfaces in doped organic semiconductors. Phys. Chem. Chem. Phys. 2020, 22, 3527–3538. 10.1039/C9CP06655A. 31994551

[ref22] Guerrini M.; Valencia A. M.; Cocchi C. Long-Range Order Promotes Charge-Transfer Excitations in Donor/Acceptor Co-Crystals. J. Phys. Chem. C 2021, 125, 20821–20830. 10.1021/acs.jpcc.1c06969.

[ref23] Andrea Rozzi C.; Maria Falke S.; Spallanzani N.; Rubio A.; Molinari E.; Brida D.; Maiuri M.; Cerullo G.; Schramm H.; Christoffers J.; Lienau C.; et al. Quantum coherence controls the charge separation in a prototypical artificial light-harvesting system. Nature Commun. 2013, 4, 1602 10.1038/ncomms2603. 23511467 PMC3615481

[ref24] Falke S. M.; Rozzi C. A.; Brida D.; Maiuri M.; Amato M.; Sommer E.; De Sio A.; Rubio A.; Cerullo G.; Molinari E.; et al. Coherent ultrafast charge transfer in an organic photovoltaic blend. Science 2014, 344, 1001–1005. 10.1126/science.1249771. 24876491

[ref25] Dorfner M. F.; Hutsch S.; Borrelli R.; Gelin M. F.; Ortmann F. Ultrafast carrier dynamics at organic donor–acceptor interfaces—a quantum-based assessment of the hopping model. J. Phys. Materials 2022, 5, 024001 10.1088/2515-7639/ac442b.

[ref26] Tamai Y.; Fan Y.; Kim V. O.; Ziabrev K.; Rao A.; Barlow S.; Marder S. R.; Friend R. H.; Menke S. M. Ultrafast long-range charge separation in nonfullerene organic solar cells. ACS Nano 2017, 11, 12473–12481. 10.1021/acsnano.7b06575. 29148715

[ref27] Chen Z.; Chen X.; Qiu B.; Zhou G.; Jia Z.; Tao W.; Li Y.; Yang Y. M.; Zhu H. Ultrafast hole transfer and carrier transport controlled by nanoscale-phase morphology in nonfullerene organic solar cells. J. Phys. Chem. Lett. 2020, 11, 3226–3233. 10.1021/acs.jpclett.0c00919. 32259443

[ref28] Bolzonello L.; Bernal-Texca F.; Gerling L. G.; Ockova J.; Collini E.; Martorell J.; van Hulst N. F. Photocurrent-Detected 2D Electronic Spectroscopy Reveals Ultrafast Hole Transfer in Operating PM6/Y6 Organic Solar Cells. J. Phys. Chem. Lett. 2021, 12, 3983–3988. 10.1021/acs.jpclett.1c00822. 33877838 PMC8154857

[ref29] De Sio A.; Lienau C. Vibronic coupling in organic semiconductors for photovoltaics. Phys. Chem. Chem. Phys. 2017, 19, 18813–18830. 10.1039/C7CP03007J. 28702561

[ref30] Tamura H.; Ramon J. G.; Bittner E. R.; Burghardt I. Phonon-Driven Exciton Dissociation at Donor- Acceptor Polymer Heterojunctions: Direct versus Bridge-Mediated Vibronic Coupling Pathways. J. Phys. Chem. B 2008, 112, 495–506. 10.1021/jp077270p. 18081341

[ref31] Polkehn M.; Tamura H.; Burghardt I. Impact of charge-transfer excitons in regioregular polythiophene on the charge separation at polythiophene-fullerene heterojunctions. J. Phys. B 2018, 51, 014003 10.1088/1361-6455/aa93d0.

[ref32] Binder R.; Lauvergnat D.; Burghardt I. Conformational dynamics guides coherent exciton migration in conjugated polymer materials: First-principles quantum dynamical study. Phys. Rev. Lett. 2018, 120, 227401 10.1103/PhysRevLett.120.227401. 29906150

[ref33] Popp W.; Polkehn M.; Binder R.; Burghardt I. Coherent charge transfer exciton formation in regioregular P3HT: A quantum dynamical study. J. Phys. Chem. Lett. 2019, 10, 3326–3332. 10.1021/acs.jpclett.9b01105. 31135165

[ref34] Peng W.-T.; Brey D.; Giannini S.; Dell’Angelo D.; Burghardt I.; Blumberger J. Exciton Dissociation in a Model Organic Interface: Excitonic State-Based Surface Hopping versus Multiconfigurational Time-Dependent Hartree. J. Phys. Chem. Lett. 2022, 13, 7105–7112. 10.1021/acs.jpclett.2c01928. 35900333 PMC9376959

[ref35] Hoffman D. P.; Ellis S. R.; Mathies R. A. Characterization of a conical intersection in a charge-transfer dimer with two-dimensional time-resolved stimulated Raman spectroscopy. J. Phys. Chem. A 2014, 118, 4955–4965. 10.1021/jp5041986. 24932925

[ref36] Ellis S. R.; Hoffman D. P.; Park M.; Mathies R. A. Difference bands in time-resolved femtosecond stimulated Raman spectra of photoexcited intermolecular electron transfer from chloronaphthalene to tetracyanoethylene. J. Phys. Chem. A 2018, 122, 3594–3605. 10.1021/acs.jpca.8b00318. 29558802

[ref37] Runge E.; Gross E. K. Density-functional theory for time-dependent systems. Phys. Rev. Lett. 1984, 52, 997 10.1103/PhysRevLett.52.997.

[ref38] Marques M. A.; Castro A.; Bertsch G. F.; Rubio A. octopus: a first-principles tool for excited electron–ion dynamics. Comput. Phys. Commun. 2003, 151, 60–78. 10.1016/S0010-4655(02)00686-0.

[ref39] Rozzi C. A.; Troiani F.; Tavernelli I. Quantum modeling of ultrafast photoinduced charge separation. J. Phys.: Condens. Matter. 2018, 30, 013002 10.1088/1361-648X/aa948a. 29047450

[ref40] Marx D.; Hutter J. Ab Initio Molecular Dynamics: Basic Theory and Advanced Methods; Cambridge University Press, 2009.

[ref41] Nose S. A unified formulation of the constant temperature molecular dynamics methods. J. Chem. Phys. 1984, 81, 511–519. 10.1063/1.447334.

[ref42] Hoover W. G. Canonical dynamics: Equilibrium phase-space distributions. Phys. Rev. A 1985, 31, 1695–1697. 10.1103/PhysRevA.31.1695. 9895674

[ref43] Hünenberger P. H. In Advanced Computer Simulation: Approaches for Soft Matter Sciences I; Dr. Holm C., Prof. Dr Kremer K., Eds.; Springer Berlin Heidelberg: Berlin, Heidelberg, 2005; pp 105–149.

[ref44] Braun E.; Moosavi S. M.; Smit B. Anomalous effects of velocity rescaling algorithms: the flying ice cube effect revisited. J. Chem. Theory. Comput. 2018, 14, 5262–5272. 10.1021/acs.jctc.8b00446. 30075070

[ref45] Barbatti M.; Sen K. Effects of different initial condition samplings on photodynamics and spectrum of pyrrole. Int. J. Quantum Chem. 2016, 116, 762–771. 10.1002/qua.25049.

[ref46] Casida M. E. Recent Advances In Density Functional Methods Part 1; World Scientific, 1995; pp 155–192.

[ref47] Casida M. E. Top. Curr. Chem.; Elsevier, 1996; Vol. 4, pp 391–439.

[ref48] Blum V.; Gehrke R.; Hanke F.; Havu P.; Havu V.; Ren X.; Reuter K.; Scheffler M. Ab initio molecular simulations with numeric atom-centered orbitals. Comput. Phys. Commun. 2009, 180, 2175–2196. 10.1016/j.cpc.2009.06.022.

[ref49] Shang H.; Raimbault N.; Rinke P.; Scheffler M.; Rossi M.; Carbogno C. All-electron, real-space perturbation theory for homogeneous electric fields: theory, implementation, and application within DFT. New. J. Phys. 2018, 20, 073040 10.1088/1367-2630/aace6d.

[ref50] Tancogne-Dejean N.; Oliveira M. J. T.; Andrade X.; Appel H.; Borca C. H.; Le Breton G.; Buchholz F.; Castro A.; Corni S.; Correa A. A.; et al. Octopus, a computational framework for exploring light-driven phenomena and quantum dynamics in extended and finite systems. J. Chem. Phys. 2020, 152, 124119 10.1063/1.5142502. 32241132

[ref51] Bitzek E.; Koskinen P.; Gähler F.; Moseler M.; Gumbsch P. Structural Relaxation Made Simple. Phys. Rev. Lett. 2006, 97, 170201 10.1103/PhysRevLett.97.170201. 17155444

[ref52] Hartwigsen C.; Gœdecker S.; Hutter J. Relativistic separable dual-space Gaussian pseudopotentials from H to Rn. Phys. Rev. B 1998, 58, 3641 10.1103/PhysRevB.58.3641. 9986014

[ref53] Goedecker S.; Teter M.; Hutter J. Separable dual-space Gaussian pseudopotentials. Phys. Rev. B 1996, 54, 1703 10.1103/PhysRevB.54.1703. 9986014

[ref54] Perdew J. P.; Wang Y. Accurate and simple analytic representation of the electron-gas correlation energy. Phys. Rev. B 1992, 45, 13244–13249. 10.1103/PhysRevB.45.13244. 10001404

[ref55] Krumland J.; Valencia A. M.; Pittalis S.; Rozzi C. A.; Cocchi C. Understanding real-time time-dependent density-functional theory simulations of ultrafast laser-induced dynamics in organic molecules. J. Chem. Phys. 2020, 153, 054106 10.1063/5.0008194. 32770886

[ref56] Raghunathan S.; Nest M. Critical Examination of Explicitly Time-Dependent Density Functional Theory for Coherent Control of Dipole Switching. J. Chem. Theory. Comput. 2011, 7, 2492–2497. 10.1021/ct200270t. 26606623

[ref57] Fuks J. I.; Helbig N.; Tokatly I. V.; Rubio A. Nonlinear phenomena in time-dependent density-functional theory: What Rabi oscillations can teach us. Phys. Rev. B 2011, 84, 075107 10.1103/PhysRevB.84.075107.

[ref58] Fuks J. I.; Maitra N. T. Challenging adiabatic time-dependent density functional theory with a Hubbard dimer: the case of time-resolved long-range charge transfer. Phys. Chem. Chem. Phys. 2014, 16, 14504–14513. 10.1039/C4CP00118D. 24643509

[ref59] Polkehn M.; Eisenbrandt P.; Tamura H.; Burghardt I. Quantum dynamical studies of ultrafast charge separation in nanostructured organic polymer materials: Effects of vibronic interactions and molecular packing. Int. J. Quantum Chem. 2018, 118, e25502 10.1002/qua.25502.

[ref60] Yabana K.; Bertsch G. Time-dependent local-density approximation in real time. Phys. Rev. B 1996, 54, 4484 10.1103/PhysRevB.54.4484. 9986402

[ref61] Castro A.; Marques M. A.; Rubio A. Propagators for the time-dependent Kohn–Sham equations. J. Chem. Phys. 2004, 121, 3425–3433. 10.1063/1.1774980. 15303905

[ref62] Börzsönyi Á.; Heiner Z.; Kovács A.; Kalashnikov M.; Osvay K. Measurement of pressure dependent nonlinear refractive index of inert gases. Org. Electron. 2010, 18, 25847–25854. 10.1364/OE.18.025847. 21164930

[ref63] Picón A.; Biegert J.; Jaron-Becker A.; Becker A. Coherent control of the vibrational state population in a nonpolar molecule. Phys. Rev. A 2011, 83, 023412 10.1103/PhysRevA.83.023412.

[ref64] De Giovannini U.; Brunetto G.; Castro A.; Walkenhorst J.; Rubio A. Simulating Pump–Probe Photoelectron and Absorption Spectroscopy on the Attosecond Timescale with Time-Dependent Density Functional Theory. ChemPhysChem 2013, 14, 1363–1376. 10.1002/cphc.201201007. 23520148

[ref65] Yamada S.; Noda M.; Nobusada K.; Yabana K. Time-dependent density functional theory for interaction of ultrashort light pulse with thin materials. Phys. Rev. B 2018, 98, 245147 10.1103/PhysRevB.98.245147.

[ref66] Zhang X.; Wang F.; Liu Z.; Feng X.; Pang S. Controlling energy transfer from intense ultrashort light pulse to crystals: A comparison study in attosecond and femtosecond regimes. Phys. Lett. A 2020, 384, 126710 10.1016/j.physleta.2020.126710.

[ref67] Jacobs M.; Krumland J.; Valencia A. M.; Wang H.; Rossi M.; Cocchi C. Ultrafast charge transfer and vibronic coupling in a laser-excited hybrid inorganic/organic interface. Adv. Phys: X 2020, 5, 1749883 10.1080/23746149.2020.1749883.

[ref68] Jacobs M.; Krumland J.; Cocchi C. Laser-Controlled Charge Transfer in a Two-Dimensional Organic/Inorganic Optical Coherent Nanojunction. ACS Appl. Nano Mater. 2022, 5, 5187–5195. 10.1021/acsanm.2c00253.

[ref69] Negre C. F.; Fuertes V. C.; Oviedo M. B.; Oliva F. Y.; Sánchez C. G. Quantum dynamics of light-induced charge injection in a model dye–nanoparticle complex. J. Phys. Chem. C 2012, 116, 14748–14753. 10.1021/jp210248k.

[ref70] Oviedo M. B.; Zarate X.; Negre C. F.; Schott E.; Arratia-Pérez R.; Sánchez C. G. Quantum dynamical simulations as a tool for predicting photoinjection mechanisms in dye-sensitized TiO2 solar cells. J. Phys. Chem. Lett. 2012, 3, 2548–2555. 10.1021/jz300880d. 26295873

[ref71] Domínguez-Castro A.; Frauenheim T. Impact of vibronic coupling effects on light-driven charge transfer in pyrene-functionalized middle and large-sized metalloid gold nanoclusters from Ehrenfest dynamics. Phys. Chem. Chem. Phys. 2021, 23, 17129–17133. 10.1039/D1CP02890A. 34355230

[ref72] Kühne T. D.; Iannuzzi M.; Del Ben M.; Rybkin V. V.; Seewald P.; Stein F.; Laino T.; Khaliullin R. Z.; Schütt O.; Schiffmann F.; et al. CP2K: An electronic structure and molecular dynamics software package - Quickstep: Efficient and accurate electronic structure calculations. J. Chem. Phys. 2020, 152, 194103 10.1063/5.0007045. 33687235

[ref73] VandeVondele J.; Hutter J. Gaussian basis sets for accurate calculations on molecular systems in gas and condensed phases. J. Chem. Phys. 2007, 127, 114105 10.1063/1.2770708. 17887826

[ref74] Rossi M. Progress and challenges in ab initio simulations of quantum nuclei in weakly bonded systems. J. Chem. Phys. 2021, 154, 170902 10.1063/5.0042572. 34241065

[ref75] Krumland J.; Jacobs M.; Cocchi C. Ab initio simulation of laser-induced electronic and vibrational coherence. Phys. Rev. B 2022, 106, 144304 10.1103/PhysRevB.106.144304.

[ref76] Bader R. F. Atoms in Molecules - A Quantum Theory; Oxford University Press, 1990.

[ref77] Zhu L.; Kim E.-G.; Yi Y.; Bredas J.-L. Charge transfer in molecular complexes with 2, 3, 5, 6-tetrafluoro-7, 7, 8, 8-tetracyanoquinodimethane (F4-TCNQ): A density functional theory study. Chem. Mater. 2011, 23, 5149–5159. 10.1021/cm201798x.

[ref78] Krumland J.; Valencia A. M.; Cocchi C. Exploring organic semiconductors in solution: the effects of solvation, alkylization, and doping. Phys. Chem. Chem. Phys. 2021, 23, 4841–4855. 10.1039/D0CP06085B. 33605967

[ref79] Zwickl J.; Shenvi N.; Schmidt J. R.; Tully J. C. Transition State Barriers in Multidimensional Marcus Theory. J. Phys. Chem. A 2008, 112, 10570–10579. 10.1021/jp805065g. 18826200

[ref80] Ren H.-S.; Ming M.-J.; Ma J.-Y.; Li X.-Y. Theoretical Calculation of Reorganization Energy for Electron Self-Exchange Reaction by Constrained Density Functional Theory and Constrained Equilibrium Thermodynamics. J. Phys. Chem. A 2013, 117, 8017–8025. 10.1021/jp4046935. 23895675

[ref81] Matyushov D. V. Reorganization energy of electron transfer. Phys. Chem. Chem. Phys. 2023, 25, 7589–7610. 10.1039/D2CP06072H. 36876860

[ref82] Mansour A. E.; Valencia A. M.; Lungwitz D.; Wegner B.; Tanaka N.; Shoji Y.; Fukushima T.; Opitz A.; Cocchi C.; Koch N. Understanding the evolution of the Raman spectra of molecularly p-doped poly (3-hexylthiophene-2, 5-diyl): signatures of polarons and bipolarons. Phys. Chem. Chem. Phys. 2022, 24, 3109–3118. 10.1039/D1CP04985B. 35040854

[ref83] Herperger K. R.; Krumland J.; Cocchi C. Laser-Induced Electronic and Vibronic Dynamics in the Pyrene Molecule and Its Cation. J. Phys. Chem. A 2021, 125, 9619–9631. 10.1021/acs.jpca.1c06538. 34714646

[ref84] Parandekar P. V.; Tully J. C. Detailed Balance in Ehrenfest Mixed Quantum-Classical Dynamics. J. Chem. Theory. Comput. 2006, 2, 229–235. 10.1021/ct050213k. 26626509

[ref85] Liu W.-H.; Wang Z.; Chen Z.-H.; Luo J.-W.; Li S.-S.; Wang L.-W. Algorithm advances and applications of time-dependent first-principles simulations for ultrafast dynamics. Wiley Interdiscip. Rev. Comput. Mol. Sci. 2022, 12, e1577 10.1002/wcms.1577.

[ref86] Alimi R.; García-Vela A.; Gerber R. B. A remedy for zero-point energy problems in classical trajectories: A combined semiclassical/classical molecular dynamics algorithm. J. Chem. Phys. 1992, 96, 2034–2038. 10.1063/1.462106.

[ref87] Shu Y.; Dong S. S.; Parker K. A.; Bao J. L.; Zhang L.; Truhlar D. G. Extended Hamiltonian molecular dynamics: semiclassical trajectories with improved maintenance of zero point energy. Phys. Chem. Chem. Phys. 2018, 20, 30209–30218. 10.1039/C8CP04914A. 30489584

[ref88] Mukherjee S.; Barbatti M. A Hessian-Free Method to Prevent Zero-Point Energy Leakage in Classical Trajectories. J. Chem. Theory. Comput. 2022, 18, 4109–4116. 10.1021/acs.jctc.2c00216. 35679615

[ref89] You P.; Xu J.; Lian C.; Zhang C.; Li X.-Z.; Wang E.-G.; Meng S. Quantum dynamics simulations: combining path integral nuclear dynamics and real-time TDDFT. Electron. Struct. 2019, 1, 044005 10.1088/2516-1075/ab58fc.

