# Supplementary material for: Pulse-Induced Dynamics of a Charge-Transfer Complex from First Principles
Source: J Phys Chem A. 2023 Oct 12;127(42):8794–805. doi: 10.1021/acs.jpca.3c03709 (PMC10614200; doi:10.1021/acs.jpca.3c03709)
Supplement: Supplementary file 1 — jp3c03709_si_001.pdf [file jp3c03709_si_001.pdf]

SUPPORTING INFORMATION

Pulse-Induced Dynamics of a Charge-Transfer Complex from First Principles

Matheus Jacobs,*,† Jannis Krumland,† Ana M. Valencia,†,‡ and Caterina Cocchi*,†,‡,¶

†*Physics Department and IRIS Adlershof, Humboldt-Universität zu Berlin, 12489 Berlin, Germany*

‡*Institute of Physics, Carl von Ossietzky Universität Oldenburg, 26129 Oldenburg, Germany*

¶*Center for Nanoscale Dynamics (CeNaD), Carl von Ossietzky Universität, 26129 Oldenburg, Germany*

E-mail: jacobs@physik.hu-berlin.de; caterina.cocchi@uni-oldenburg.de

Additional Details on the Theoretical Background

Kinetic Energy of Vibrational Modes

Assuming the harmonic approximation for the nuclear motion, the potential energy associated with the nuclear configuration

$$R = \{\mathbf{R}_1, \ldots, \mathbf{R}_M\} = R_0 + \Delta R, \tag{1}$$

where R_0 is the equilibrium configuration and ΔR represents a small deviation from it, can be written as

$$E^{\text{harm}}(R) = E(R_0) + \frac{1}{2} \sum_{J,I=1}^{M} \sum_{\mu,\nu=1}^{3} K_{J\mu,I\nu} \Delta R_{J\mu} \Delta R_{I\nu}, \tag{2}$$

where the generalized spring constant

$$K_{J\mu,I\nu} = \left. \frac{\partial^2 E(R)}{\partial R_{J\mu} \partial R_{I\nu}} \right|_{R=R_0} \tag{3}$$

is a matrix whose components can be obtained from first principles using density functional perturbation theory. Diagonalizing this matrix yields $3M$ decoupled modes as rows of the orthogonal transformation matrix $T_{\alpha,J\mu}$, together with the corresponding eigenfrequencies ω_α. Six of these $3M$ modes do not describe vibrations, but translations and rotations of the whole system. The normal coordinates $\mathcal{Q}_\alpha$ associated with these modes follow the trajectories

$$\mathcal{Q}_\alpha(t) = \sum_{J,\mu} T_{\alpha,J\mu} R_{J\mu}(t) \approx \mathcal{Q}_\alpha^{(0)} \cos(\omega_\alpha t), \tag{4}$$

within the range of validity of the harmonic approximation. The normal coordinates are mass-weighted, such that the rows of the transformation matrix $T_{\alpha,J\mu}$ do not represent actual

displacements. The potential energy becomes

$$E^{\text{harm}}(R(\mathcal{Q})) = E(R_0) + \frac{1}{2}\sum_{\alpha} \omega_\alpha^2 \mathcal{Q}_\alpha^2. \tag{5}$$

The accuracy of the individual harmonic potential energy terms, $\omega_\alpha^2 \mathcal{Q}_\alpha^2/2$, hinge on the validity of the harmonic approximation. Hence, we instead monitor the corresponding kinetic energies in time, which are simply given as

$$E_\alpha^{\text{kin}}(t) = \dot{\mathcal{Q}}_\alpha^2(t)/2. \tag{6}$$

This remains valid for anharmonic motion; in this case, the transformation from cartesian coordinates to normal modes can be seen as a change into a better, albeit not optimal, basis to represent the nuclear motion.

Analysis of Vibronic Effects

As seen in Eq. 3, the forces acting on the nuclei due to the electrons are given as

$$\mathbf{F}_J^{\text{el}}(t) = \frac{\partial}{\partial \mathbf{R}_J} \int \mathrm{d}^3 r\, n(\mathbf{r}, t) v_{\text{en}}(\mathbf{r}, R(t)). \tag{7}$$

The integral is the negative of the electrostatic energy between the set of nuclear point charges with coordinates $R(t) = \{\mathbf{R}_1(t), \ldots, \mathbf{R}_N(t)\}$ and the continuous electronic charge density $-n(\mathbf{r}, t)$. Thus, the gradient with respect to $\mathbf{R}_J$ is the electrostatic force exerted by the electronic cloud onto nucleus J. For interpretation purposes, it is useful to partition this force into two different parts. To this end, we expand the many-body state $|\Psi(t)\rangle$ with electron density $n(\mathbf{r}, t)$ in the adiabatic basis:

$$|\Psi(t)\rangle = \sum_j c_j(t) e^{-iE_j(R(t))t} \left| j; R(t) \right\rangle, \tag{8}$$

where $c_j(t)$ is the superposition coefficient belonging to the electronic eigenstate $|j; R(t)\rangle$ for the nuclear configuration $R(t)$, with energy $E_j(R(t))$. Using this representation, the electron density can be expressed as

$$n(\mathbf{r}, t) = \langle\Psi(t)|\, \hat{n}(\mathbf{r})\, |\Psi(t)\rangle = n^{\mathrm{pop}}(\mathbf{r}, t) + n^{\mathrm{coh}}(\mathbf{r}, t), \tag{9}$$

where $\hat{n}(\mathbf{r}) = \sum_k \delta(\mathbf{r} - \hat{\mathbf{r}}_k)$ is the electron density operator and

$$n^{\mathrm{pop}}(\mathbf{r}, t) = \sum_i |c_i(t)|^2 n_i(\mathbf{r}; R(t)) \tag{10a}$$

$$n^{\mathrm{coh}}(\mathbf{r}, t) = \sum_{i \neq f} c_f^*(t) c_i(t) e^{i\Omega_{if}(t)t} n_{if}(\mathbf{r}; R(t)). \tag{10b}$$

Here, $n_i(\mathbf{r}; R(t)) = \langle i; R(t)|\, \hat{n}(\mathbf{r})\, |i; R(t)\rangle$ is the electron density of state i, $n_{if}(\mathbf{r}; R(t)) = \langle f; R(t)|\, \hat{n}(\mathbf{r})\, |i; R(t)\rangle$ stands for the transition density of the transition from the initial state i to the final state f, and $\Omega_{if}(t)$=$E_f(R(t))$-$E_i(R(t))$ is the related transition frequency. $\Omega_{if}(t)$ changes slightly on the time scale of the nuclear motion, shifting with respect to the initial value due to the combination of the (static) Stokes' shift and vibrationally coherent variations.[1–3] The superscripts of the densities in Eqs. (10a) and (10b) indicate that they are related to the populations $|c_i(t)|^2$ and the coherences $c_f^*(t)c_i(t)e^{i\Omega_{if}(t)t}$ of the system, respectively, *i.e.* the diagonal and off-diagonal components of the electronic density matrix.

The combination of Eq. (7) with Eqs. (10a) and (10b) yields two forces[4]

$$\mathbf{F}_J^{\mathrm{el}}(t) = \mathbf{F}_J^{\mathrm{pop}}(t) + \mathbf{F}_J^{\mathrm{coh}}(t), \tag{11}$$

which are quite distinct in nature. The laser pulse excites the system, increasing the coefficients $c_n(t)$ of the excited states. After the corresponding pulse is over, they remain approximately constant for a while, assuming that no further strong non-adiabatic coupling between potential-energy surfaces (PES) is present on this short timescale. Thus, the population-related force $\mathbf{F}_J^{\mathrm{pop}}(t)$ strongly increases during irradiation, but stays nearly con-

stant afterwards. It is thus effectively a static force, switched on by the laser, and reflects a sudden displacement of the average PES, on which the motion occurs in the Ehrenfest scheme [Fig. S2a)]. This displacement is due to the mixing in of excited-state PES,[5] which generally have minima at different positions. Thus, the system, still residing at the position of the ground-state minimum, is put in an unstable state: The modes start to oscillate around the new minimum with their respective eigenfrequencies. These frequencies are close to the ground-state ones if the system is not excited too strongly, *i.e.* the excited-state PES are not sufficiently present in the average PES to imprint their curvatures on it.

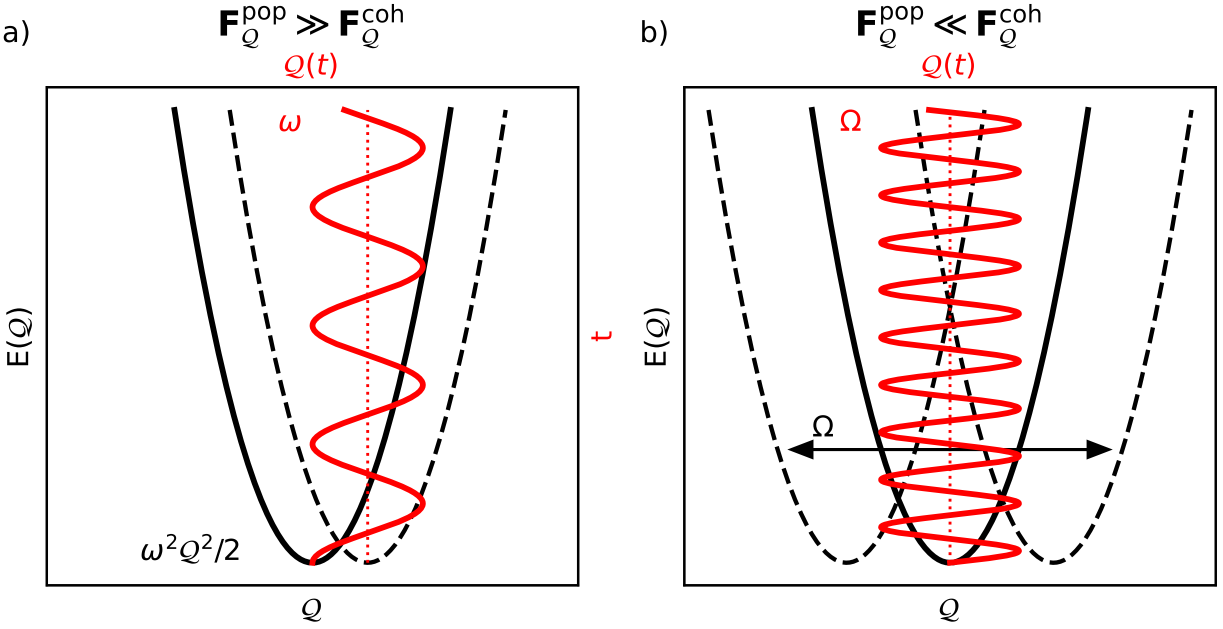

Figure S1: Schematic representation of the ground-state potential energy (solid black) and the average potential energy after laser irradiation (dashed black) as a function of the normal coordinate $\mathcal{Q}$. The red curves show the $\mathcal{Q}(t)$ trajectories. The corresponding period of oscillation is the eigenfrequency ω_0 of the harmonic oscillator, if the population-related force is dominant (a), or the electronic transition frequency Ω, if the coherent force is more significant (b).

The coherence-related force $\mathbf{F}_J^{\mathrm{coh}}(t)$, on the other hand, is dynamical; it has an additional periodic time dependence and oscillates at the electronic transition frequencies $\Omega_{if}(t)$. In the present case, the transition frequencies are much higher than the vibrational resonances of the system. Thus, the periodic forces $\mathbf{F}_J^{\mathrm{coh}}(t)$ drive a fast off-resonance oscillation of some modes $\mathcal{Q}_\alpha$, if the involved electronic transition densities have the proper spatial profile to

exert a net electrostatic force,

$$F_{\alpha}^{\mathrm{coh}}(t) = \sum_{J} \sum_{\mu} \frac{T_{\alpha,J\mu}}{M_J} F_{J\mu}^{\mathrm{coh}}(t). \tag{12}$$

If these forces are strong, they can drive a remarkable oscillation.

Additional First-Principle Results

Frontier Orbitals

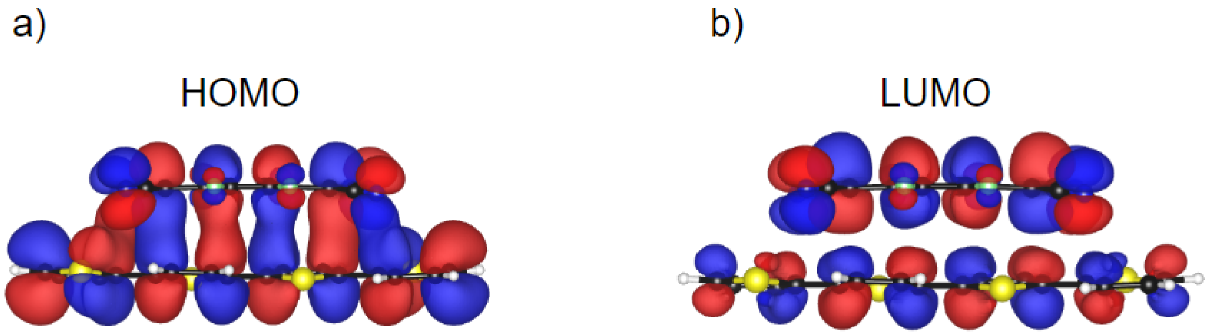

Figure S2: a) Highest occupied molecular orbital (HOMO) and b) lowest unoccupied molecular orbital (LUMO) of the F4TCNQ-4T complex. The bonding (anti-bonding) character of the HOMO (LUMO) should be noticed.

Ground-state normal modes

Vibrational normal modes are calculated using FHI-aims code,[6,7] in the Perdew-Burke-Ernzerhof (PBE) approximation[8] for v_{xc} and including pairwise van der Waals corrections.[9] Tight integration grids and TIER2 basis sets[10] were adopted. The atomic positions were relaxed until the Hellmann-Feynman forces are smaller than 10^{-4} eV/Å.

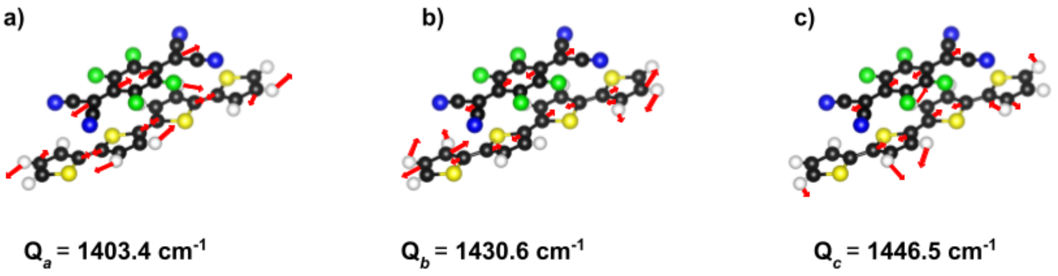

Figure S3: Coupled vibrational modes a) $\mathcal{Q}_a$, b) $\mathcal{Q}_b$, and c) $\mathcal{Q}_c$ in the 4T-F4TCNQ complex.

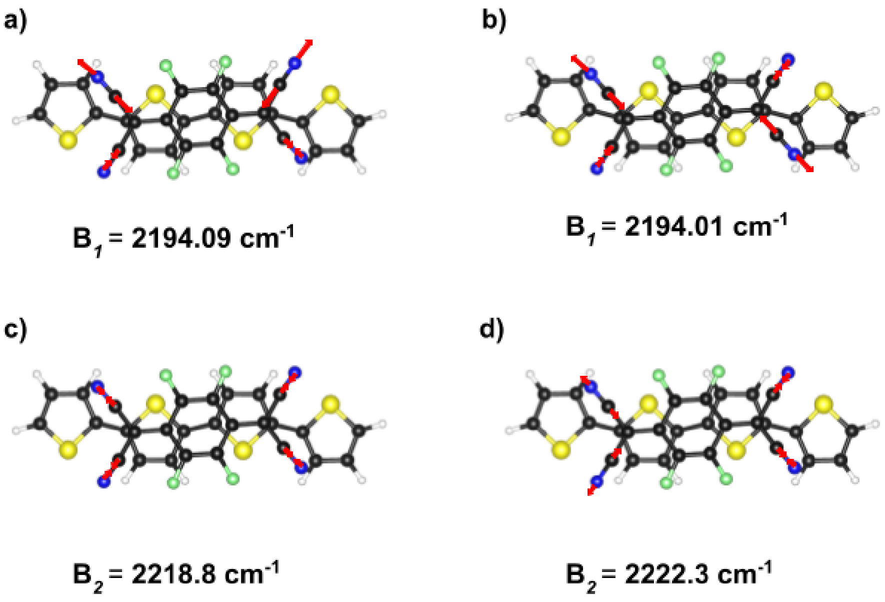

Figure S4: Visualization of the C≡N modes a) and b) $\mathcal{B}_1$, and c)-d) $\mathcal{B}_2$.

Time- and Frequency-Resolved Dipole Moments

The time- and frequency-resolved dipole moment (dipole power)[11,12] is a powerful tool to monitor the dynamical state population in both linear and non-linear regimes. This quantity provides a connection between coherences and laser-induced population transfer. The dipole moment is defined as

$$\mathbf{d}(t) = -e \int d^3r \mathbf{r} \rho(\mathbf{r}, t) + e \sum_K Z_K \mathbf{R}_K(t), \tag{13}$$

where Z_K is the atomic number of the K-th nucleus and $\mathbf{R}_K$ its coordinate. Applying a short-time Fourier transform and averaging, we obtain the dipole power as:

$$\mathbf{d}(t,\omega) = \left\langle \left| \int dt' e^{-i\omega t'} w(t-t')\mathbf{d}(t') \right| \right\rangle , \tag{14}$$

where w is a Gaussian window function. As depicted in Figure S5a,c,e, the dipole response of 4T-F4TCNQ at finite temperatures is almost completely centered around the frequency of E1 at 1.2 eV with a small spread to other frequency components[12] at 2.5 eV, which match the HOMO → LUMO excitation from the 4T. In this case, this behavior can be attributed to the donor and acceptor being driven apart in time at finite temperatures (Figure S5b,d,f), which contributes to the decrease of charge transfer compared to the ground-state reference value.

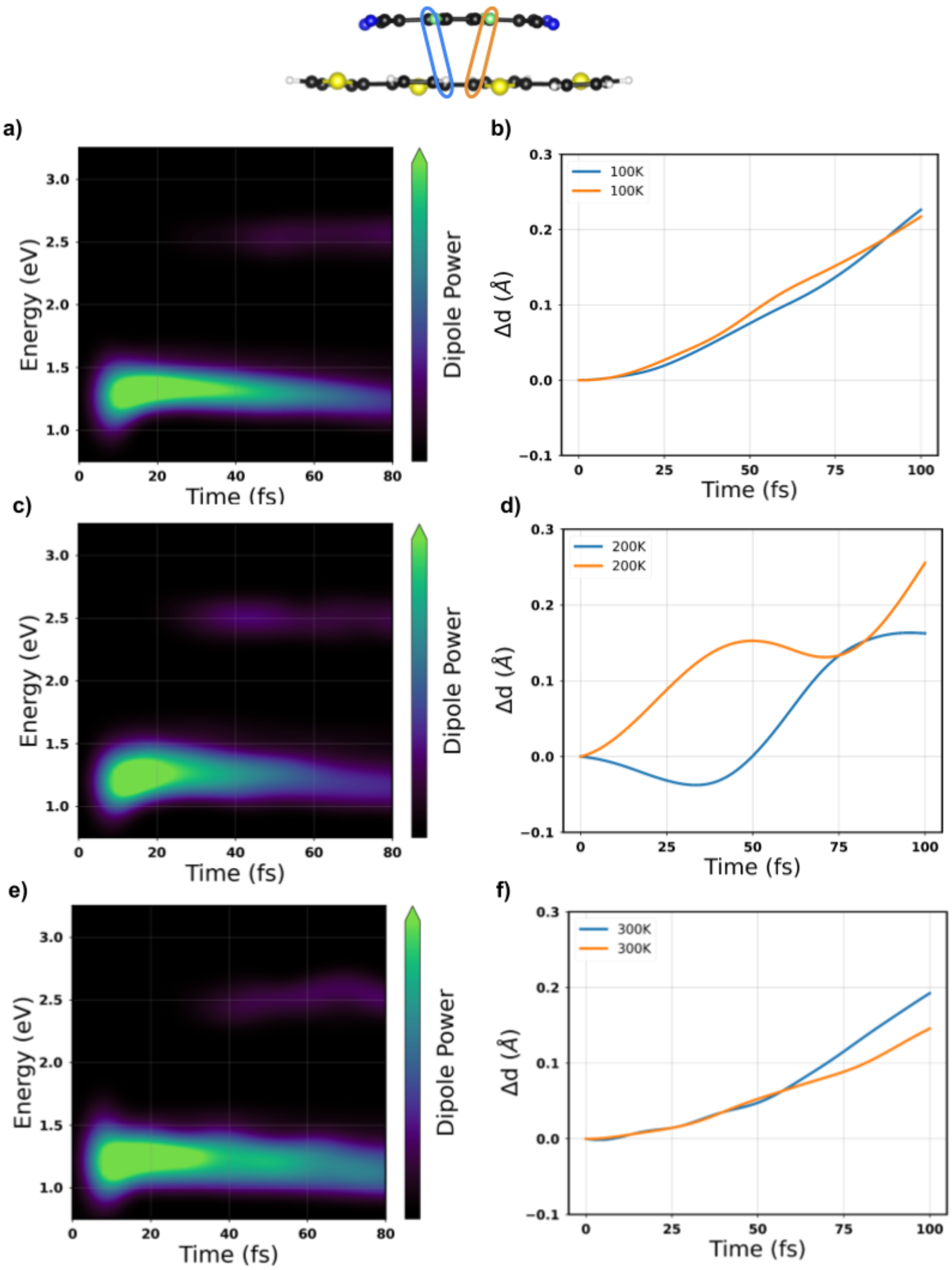

Figure S5: (a,c,e) Dipole power and (b,d,f) variation of the distance between the donor and acceptor molecule at each finite temperature value taken as the separation between central carbon atoms in F4TCNQ and 4T, depicted by blue and orange circles on top.

References

(1) Bonafe, F. P.; Hernández, F. J.; Aradi, B.; Frauenheim, T.; Sánchez, C. G. Fully Atomistic Real-Time Simulations of Transient Absorption Spectroscopy. *J. Phys. Chem. Lett.* **2018**, *9*, 4355–4359, PMID: 30024765.

(2) Hernández, F. J.; Bonafé, F. P.; Aradi, B.; Frauenheim, T.; Sánchez, C. G. Simulation of Impulsive Vibrational Spectroscopy. *J. Phys. Chem. A* **2019**, *123*, 2065–2072, PMID: 30767532.

(3) Krumland, J.; Valencia, A. M.; Pittalis, S.; Rozzi, C. A.; Cocchi, C. Understanding real-time time-dependent density-functional theory simulations of ultrafast laser-induced dynamics in organic molecules. *J. Chem. Phys.* **2020**, *153*, 054106.

(4) Agostini, F. An exact-factorization perspective on quantum-classical approaches to excited-state dynamics. *Eur. Phys. J. B* **2018**, *91*, 143.

(5) Prezhdo, O. V. Mean field approximation for the stochastic Schrödinger equation. *J. Chem. Phys.* **1999**, *111*, 8366–8377.

(6) Blum, V.; Gehrke, R.; Hanke, F.; Havu, P.; Havu, V.; Ren, X.; Reuter, K.; Scheffler, M. Ab initio molecular simulations with numeric atom-centered orbitals. *Comput. Phys. Commun.* **2009**, *180*, 2175 – 2196.

(7) Shang, H.; Raimbault, N.; Rinke, P.; Scheffler, M.; Rossi, M.; Carbogno, C. All-electron, real-space perturbation theory for homogeneous electric fields: theory, implementation, and application within DFT. *New. J. Phys.* **2018**, *20*, 073040.

(8) Perdew, J. P.; Burke, K.; Ernzerhof, M. Generalized Gradient Approximation Made Simple. *Phys. Rev. Lett.* **1996**, *77*, 3865–3868.

(9) Tkatchenko, A.; Scheffler, M. Accurate Molecular Van Der Waals Interactions from

Ground-State Electron Density and Free-Atom Reference Data. *Phys. Rev. Lett.* **2009**, *102*, 073005.

(10) Havu, V.; Blum, V.; Havu, P.; Scheffler, M. Efficient O(N) integration for all-electron electronic structure calculation using numeric basis functions. *J. Comp. Phys.* **2009**, *228*, 8367 – 8379.

(11) Kuda-Singappulige, G. U.; Wildman, A.; Lingerfelt, D. B.; Li, X.; Aikens, C. M. Ultrafast Nonradiative Decay of a Dipolar Plasmon-like State in Naphthalene. *J. Phys. Chem. A* **2020**, *124*, 9729–9737, PMID: 33181013.

(12) Krumland, J.; Jacobs, M.; Cocchi, C. Ab initio simulation of laser-induced electronic and vibrational coherence. *Phys. Rev. B* **2022**, *106*, 144304.
